# Multiplatform Metabolomics Characterization Reveals Novel Metabolites and Phospholipid Compositional Rules of *Haemophilus influenzae* Rd KW20

**DOI:** 10.3390/ijms241311150

**Published:** 2023-07-06

**Authors:** Miguel Fernández-García, Manuel Ares-Arroyo, Emilia Wedel, Natalia Montero, Coral Barbas, Mª Fernanda Rey-Stolle, Bruno González-Zorn, Antonia García

**Affiliations:** 1 Centro de Metabolómica y Bioanálisis (CEMBIO), Facultad de Farmacia, Universidad San Pablo-CEU, CEU Universities, Urbanización Montepríncipe, 28660 Boadilla del Monte, Spain; miguel.fernandezgarcia@ceu.es (M.F.-G.);; 2 Departamento de Ciencias Médicas Básicas, Facultad de Medicina, Universidad San Pablo-CEU, CEU Universities, Urbanización Montepríncipe, 28660 Boadilla del Monte, Spain; 3 Antimicrobial Resistance Unit (ARU), Departamento de Sanidad Animal and Centro de Vigilancia Sanitaria Veterinaria (VISAVET), Complutense University of Madrid, 28040 Madrid, Spainbgzorn@ucm.es (B.G.-Z.)

**Keywords:** microbial metabolomics, microbial lipidomics, phospholipidome, phospholipid compositional model, metabolome characterization, lipidome characterization, *Haemophilus influenzae*

## Abstract

*Haemophilus influenzae* is a gram-negative bacterium of relevant clinical interest. *H. influenzae* Rd KW20 was the first organism to be sequenced and for which a genome-scale metabolic model (GEM) was developed. However, current *H. influenzae* GEMs are unable to capture several aspects of metabolome nature related to metabolite pools. To directly and comprehensively characterize the endometabolome of *H. influenzae* Rd KW20, we performed a multiplatform MS-based metabolomics approach combining LC-MS, GC-MS and CE-MS. We obtained direct evidence of 15–20% of the endometabolome present in current *H. influenzae* GEMs and showed that polar metabolite pools are interconnected through correlating metabolite islands. Notably, we obtained high-quality evidence of 18 metabolites not previously included in *H. influenzae* GEMs, including the antimicrobial metabolite cyclo(Leu-Pro). Additionally, we comprehensively characterized and evaluated the quantitative composition of the phospholipidome of *H. influenzae*, revealing that the fatty acyl chain composition is largely independent of the lipid class, as well as that the probability distribution of phospholipids is mostly related to the conditional probability distribution of individual acyl chains. This finding enabled us to provide a rationale for the observed phospholipid profiles and estimate the abundance of low-level species, permitting the expansion of the phospholipidome characterization through predictive probabilistic modelling.

## 1. Introduction

*Haemophilus influenzae* is a gram-negative, pleomorphic bacillus that belongs to the Gammaproteobacteria class. Attending to the presence and type of its polysaccharide capsule, this species is classified into serotypes a–f and unencapsulated serotypes, which are also referred to as non–typeable *H. influenzae* (NTHi). All serotypes are capable of infecting humans and establishing disease, with serotype b (Hib) being the most virulent [1,2]. *H. influenzae* and, in particular, Hib cause a wide spectrum of clinical manifestations, especially in children. These complications range from otitis media and bronchitis to more severe invasive conditions, such as pneumonia, meningitis, and sepsis [3]. Conjugate vaccines against Hib have significantly reduced the mortality rates attributable to *H. influenzae* in the developed world. According to a 2015 estimate [4], Hib is responsible for an annual death toll of more than 29,000 children worldwide, with most deaths occurring in low–income countries. NTHi is recently gaining attention as an underestimated pathogen, for which no current vaccine has been developed [5,6,7].

Due to its relevance from a public health perspective and the well-known techniques used for its culture in vitro [8], it is not surprising that several pioneering studies have contributed to characterizing *H. influenzae* from a systems biology perspective. The genome of *H. influenzae* was the first entire genome sequenced from a free-living organism, and it spans over 1.83 Mb and contains ~1700 genes [9]. Further advances in omic technologies allowed the performance of high-throughput gene and protein expression analyses that, while mostly aiming to perform differential expression analyses, also characterized a substantial part of the compendium of transcripts [10] and proteins expressed in *H. influenzae* [11,12,13,14].

One of the next frontiers in systems biology is the characterization of small molecules implicated in the metabolism of an organism from a global perspective, as their diverse physicochemical nature prohibits the use of amplification techniques for their determination. However, *H. influenzae* was the first organism for which a genome-scale metabolic model (GEM) was reconstructed [15], and its metabolic capabilities have been extensively studied [16,17,18]. The first GEM of *H. influenzae* inferred from gene annotation [9] consisted of an initial metabolic architecture, which contained 343 metabolites and 488 reactions. The next developed GEM, i.e., *i*CS400, contained 367 unique metabolites and 546 reactions encompassing central carbon, amino acid, nucleotide, and lipid metabolism, as well as electron transport reactions and transport reactions between the cell and its environment [19]. Recently, an updated GEM, i.e., *i*NL638, was developed, which contains 746 metabolites and 1385 reactions [18]. Significant advances in the developed GEMs include the addition of tRNA metabolism, enhanced presence of lipid species, inclusion of significant cofactors, incorporation of a periplasm compartment, extensive literature curation, and usage of more complex biomass functions [18]. However, the metabolite pool composition of *H. influenzae* is poorly described, as only a limited number of metabolomic studies have provided direct evidence of metabolites being present in *H. influenzae* [16,20,21,22]. Direct characterization of the metabolome and lipidome can address limitations in inferring metabolome qualitative composition from genomics, as metabolites whose production is not supported by the predicted enzymatic capabilities may also be detected [23]. Moreover, the qualitative composition of metabolites and lipids generated by enzymes with multiple substrate affinities cannot be inferred from simple gene annotation [24,25].

Small molecules play crucial roles not only within cells, but also in the formation of biological membranes. Similar to other gram-negative bacteria, the membrane of *H. influenzae* consists of an outer and inner membrane. Pioneering studies in other bacteria revealed that gram-negative bacterial membranes are asymmetric in nature [26,27,28] and composed of membrane proteins and glycerophospholipids (GPs), while bacterial lipopolysaccharide (LPS) predominantly conforms to the outer leaflet of the outer membrane [29,30,31]. According to the literature, the membrane of *H. influenzae* possesses one of the simplest compositions found in gram-negative bacteria, as few membrane GP classes have been found to be present in *H. influenzae* phospholipid extracts, which account for approximately 85% phosphatidylethanolamine (PE) and 15% phosphatidylglycerol (PG) relative content [32]. This relative proportion of PEs and PGs, their specific membrane locations, and the degree of unsaturation and carbon number of acyl-chain moieties are critical factors that define the properties of fluidity, membrane permeability, and stability of *H. influenzae* membranes [33,34,35]. Previous GC-MS analyses related to phospholipid hydrolysis reported that *H. influenzae* PEs contain C14:0, C16:0, C16:1, and C18:0 as predominant fatty acyl chains, while only C16:0 and C18:0 were found to be fatty acyl chains bound to PGs [36]. Several lipidomic studies revealed a notably more complex fatty acyl profile in bacterial membrane GPs [27,37,38,39]. Regarding the specific position of these fatty acyl moieties in gram-negative GPs, it is well known that fatty acyl chains bound to the sn-1 position of the glycerol backbone are preferentially saturated, while monounsaturated fatty acyl chains are preferentially found at the sn-2 position [40,41]. In addition, it is known that these unsaturations mostly correspond to the presence of Z double bonds and cyclopropane rings within fatty acyl chains [40,41]. However, the presence of low-level fatty acyl chains and the degree of lipid chain asymmetry between sn-1 and sn-2 positions of GPs have not yet been qualitatively or quantitatively addressed in *H. influenzae*.

In recent years, several ‘lipid atlases’ of distinct biological matrices have been elucidated [42]. These atlases aimed to accurately describe and quantify the lipidome found in specimens of biological origin. However, as lipid atlases are inferred from LC-MS data, they often face limitations related to sensitivity that challenge the acquisition of information from the ‘deep lipidome’. These limitations include the inability to match low-quality MS/MS spectra or determine lipid species whose abundance is under the limit of detection.

In this study, we performed a multiplatform mass-spectrometry approach that enabled us to qualitatively and quantitatively characterize the endometabolome and phospholipidome of *H. influenzae*. We identified 18 metabolite species and GPs not previously described in the reference metabolic model and elucidated the chemical structure of GPs present in *H. influenzae* to the species level. Notably, the evaluation of the phospholipidome compositional profile allowed us to develop a mathematical rationale that could be used to explain the reasons that some phospholipids predicted to occur were not observed in our lipidomic analysis and permitted the expansion of the phospholipidome by predicting the abundance order of magnitude of these low-level species.

## 2. Results and Discussion

### 2.1. Global Compositional Properties of the Experimentally Determined Metabolome of Haemophilus influenzae

**Figure 1:**
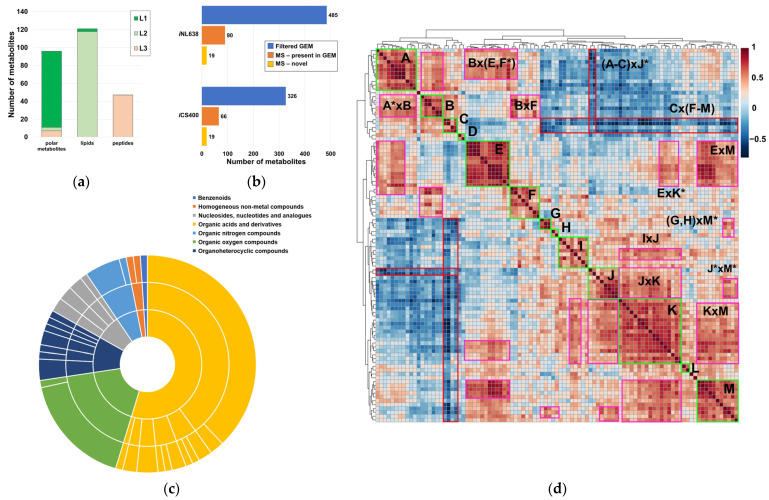
Qualitative and semiquantitative properties of the metabolome of *H. influenzae*. (**a**) Confidence in the annotation of polar metabolites, lipids, and linear peptides with unknown function; (**b**) metabolome coverage achieved via our multiplatform MS-based metabolomics approach and novel polar metabolites are annotated, both with regard to the filtered small molecule datasets obtained from *i*CS400 and *i*NL638 GEMs (each lipid type was considered to be a representative example, including the novel phospholipid subclass, LPE); (**c**) chemical ontology classification of annotated polar metabolites, excluding linear peptides of unknown function; (**d**) correlation heatmap of abundances in polar metabolites, not including linear peptides of unknown function. Self-correlating metabolite islands are denoted by letters. Intercorrelating islands are denoted by the product of letters. Asterisks (*) in the correlation heatmap specify that only part of the self-correlating island was involved in the intercorrelation.

A total of 257 chemical species with a wide variety of functional groups and polarities were annotated with varying degrees of confidence (Figure 1a). These species included 96 polar metabolites (Table S1, File S1), lipids encompassed in 64 sum compositions (with evidence for the presence of at least 118 lipids, Tables S1 and S2), and 47 linear peptides with unknown functions (Figure 1a, Table S1). Remarkably, most polar metabolites were identified with an L1 level of confidence in the annotation, except for most peptides, which had L3 annotations (Figure 1a, Table S1). Interestingly, lipid annotations showed a high level of confidence, being predominantly L2 (Figure 1a). Taken together, these annotations accounted for 15–20% of the small molecule compendium of *H. influenzae*, as captured in current GEMs (Figure 1b, Table S3). Although our study provided a modest metabolome coverage compared to the global metabolome present in current *H. influenzae* GEMs, these values were consistent with the current state-of-the art coverage of single resources in untargeted metabolomics [43]. Systematic chemical ontology analysis revealed the presence of several metabolite classes in the experimentally determined metabolome, with notable representation of peptides, lipids, and carbohydrates (Figure 1c, Table S1) attributable to the combination of techniques used in this analytical approach. Correlation analysis of metabolite pools showed the presence of 13 self-correlating metabolite islands among polar metabolites, excluding peptides with unknown function (A–M, Figure 1d, Table S1). These self-correlating islands exhibited partial intercorrelation (Figure 1d, Table S4). Very high correlation coefficients were observed for expected metabolite pairs with well-known biochemical associations, such as valine–isoleucine, glutathione disulfide–S-adenosylmethionine, or the branched-chain keto acid dehydrogenase products 3-methyl-2-oxovalerate–2-ketoisocaproate (Table S4), which demonstrate the usefulness of the correlation analysis employed. Additionally, very high self-correlations between peptides and correlations between specific lipid sum compositions and polar metabolites were observed (Table S5).

**Figure 2:**
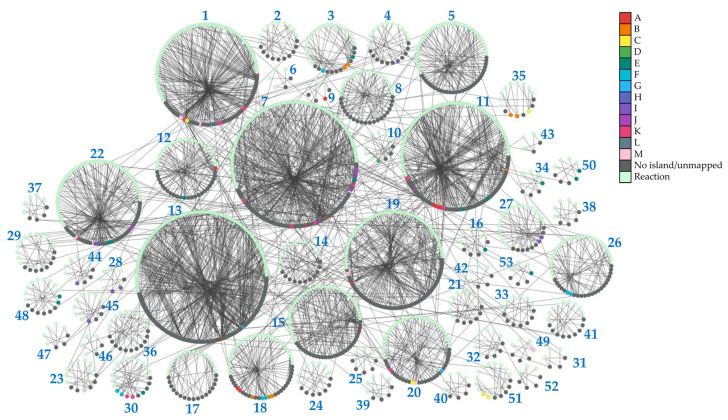
Topology of identified network clusters in the *i*NL638 metabolite–reaction subnetwork and mapping of metabolites associated with self-correlating islands. Light green nodes represent reactions; dark grey nodes represent unmapped species nodes and species not associated to correlating islands A–M; species that belong to identified metabolite self-correlation islands are represented according to the color legend.

To provide insights into the topology of *H. influenzae* metabolic pathways, we performed community clustering of the *i*NL638 periplasmic and cytoplasmic metabolite–reaction subnetwork (File S2). The clustering algorithm classified the corresponding network entities in 53 highly interconnected clusters of varying sizes and significantly enriched in different KEGG pathways (Figure 2, Table 1 and Table S6). Among these examples, several highly sized clusters (clusters 1, 7, 13, 19, 22, Figure 2, Table S6) encompassed multiple metabolic pathways (Table 1 and Table S6). Over-representation of network clusters in our experimentally determined metabolome was heterogeneous among clusters, with higher enrichment values found in clusters associated with general central carbon metabolism (cluster 1, Table 1), carbohydrate metabolism and transport (clusters 1, 3, 28, and 35, Table 1), amino acid metabolism and transport (clusters 1, 7, 9, 12, 15, 16, 18, 19, 22, 23, 30, 34, 49, 50, and 53, Table 1), nucleotide metabolism (clusters 7, 10, 11, 27, 44, and 45, Table 1), and choline transport and incorporation into lipo-oligosaccharide (cluster 48, Table 1). The absence of metabolite mapping in some large- and medium-sized clusters revealed areas of the *H. influenzae* metabolome that are poorly covered and warrant further metabolomic investigation (e.g., clusters 2, 5, 13, Figure 2, Table S6, File S2) [43]. Despite the high number of annotated lipids (Figure 1a, Tables S1 and S2), we did not observe significant enrichment in network clusters associated with lipid metabolism, as only a few of the species detected were included in previous GEMs and, therefore, could be mapped to the subnetwork metabolism (Table S3). These results highlight the need to update metabolic models that effectively capture the diversity of lipids in *H. influenzae*, as current GEMs have limited consideration for lipid composition in terms of chemical species [18,19,32]. Significant cluster enrichment of metabolite groups within the identified self-correlating islands was found (Figure 1d and Figure 2, Table S6), suggesting a relationship between high interconnection within clusters and metabolite level correlation, especially in the large-sized cluster 1 (Table 1, Figure 2). Taken together, our results provide novel and global information on the means through which the metabolite pools are inter-related in a significant part of the metabolome of *H. influenzae*.

### 2.2. Experimental Polar Metabolome Characterization Reveals Novel Metabolites of H. influenzae

Metabolome characterization unveiled the presence of 18 polar metabolites not contemplated in *i*CS400 and *i*NL638 [18,19] (Table 2 and Table S1). Notably, we identified the presence of a proline-based cyclic dipeptide not described previously in *H. influenzae*, cyclo(Leu-Pro) (Table 2 and Table S1, File S1), which was reported in other Gammaproteobacteria, such as *Pseudomonas* spp. [44,45]. Cyclo(Leu-Pro) possesses significant biological functions, including antimicrobial activity, presumably through inhibition of quorum sensing [46,47] and biofilm formation [48], suppression of pro-inflammatory cytokines (IL-1β, TNF-α, IL-6) [44], and regulation of the composition of the oral microbial consortium [49]. Hence, the production of cyclo(Leu-Pro) by *H. influenzae* may play a significant regulatory role during *H. influenzae*-associated infections and commensalism in the oropharyngeal mucosa.

Additionally, we annotated with reliability the metabolite pseudouridine, which is a post-translational modification present in various forms of RNA, including tRNA, rRNA, and mRNA [50]. We found gene annotation evidence for pseudouridine biosynthesis via tRNA pseudouridine synthases A to D (UniProt IDs: P45291, P45142, P44197, and P44039, respectively), as well as a dual-specificity RNA pseudouridine synthase (UniProt ID: P44782). These results suggest that the observed pseudouridine pool is generated from RNA degradation, despite this compound potentially being a dead-end product in *H. influenzae*, as it lacks genes known to encode for enzymes, mediating its phosphorylation and the subsequent cleavage of pseudouridine-5′-phosphate into ribose-5′-phosphate and uracil [51,52].

Moreover, our metabolomic analysis revealed the presence of high abundances of ophthalmic acid in the metabolome of *H. influenzae* (Table 2 and Table S1). Interestingly, ophthalmic acid is a poorly understood metabolite that is canonically generated via glutathione synthethases. However, these synthetases are absent in the *H. influenzae* genome, as indicated by the glutathione auxotrophy for this bacterium [53]. An additionally identified but poorly understood metabolite was *N*-methylalanine (Table 2 and Table S1, File S1). While this metabolite is known to be biosynthesized from methylamine in other bacteria via *N*-methylalanine dehydrogenase [54], no ortholog genes putatively encoding for this enzyme are present in *H. influenzae*. Further studies are needed to ascertain the possible origin and function of ophthalmic acid and *N*-methylalanine in *H. influenzae*.

Another metabolite annotated with high confidence absent in current GEMs and not supported by gene annotation evidence was polyamine cadaverine. Intriguingly, the presence of cadaverine was previously determined in *H. influenzae* via HPLC analysis [55], and lysine decarboxylase activity has been observed for several *H. influenzae* clinical isolates [56]. We also identified the metabolite 5′-methylthioadenosine, which is involved in polyamine metabolism and supported by the presence of a purine nucleoside phosphorylase and a 5′-methylthioadenosine/S-adenosylhomocysteine nucleosidase (UniProt IDs: P44552 and P45113, respectively) (Table 2 and Table S1). Another novel metabolite not supported through gene annotation and identified via metabolomics is γ-aminobutyric acid (GABA). Interestingly, the GABA subproduct succinate semialdehyde was included in *i*NL638. Thus, it may be of interest to ascertain if GABA can be biosynthesized by *H. influenzae*, despite no known orthologs putatively encoding for enzymes mediating its biosynthesis from glutamate or spermidine being found in its genome. We also detected significant abundances of the metabolite glycine betaine, which is a well-known osmoprotectant (Table 2 and Table S1), despite the absence of enzymes known to biosynthesize betaine in *H. influenzae*. Therefore, betaine may act as an additional osmoprotectant for *H. influenzae*, which was presumably acquired from the bacterial environment absorbed via the high affinity choline transporter BetT [57].

We also identified the existence of free thymine and cytosine pools not described in current GEMs that may arise from the breakdown of other nucleosides and the incorporation of carbohydrate moieties [58] (Table 2 and Table S1, File S1). We also identified two metabolite pools consisting of polyols (erythritol, ribitol, xylitol, sorbitol) and carbohydrates (trehalose, mannose, sucrose) (Table 2 and Table S1). These results were unexpected since *H. influenzae* is not capable of fermenting such compounds [59,60] and, therefore, they may represent side reactions or simple absorption and accumulation of these compounds from the media. Lastly, we annotated with high confidence the metabolite threonic acid, which was previosuly described as a degradation product from carbohydrate-like compounds [61]. Taken together, our results suggest modest differences in metabolic pathway topology than were reported in previous GEMs, which are worth for further exploration and allowed us to find unexpected metabolome constituents, which, in turn, could not be inferred solely from current gene annotation evidence.

### 2.3. Global Compositional Properties of the Phospholipidome of H. influenzae

**Figure 3:**
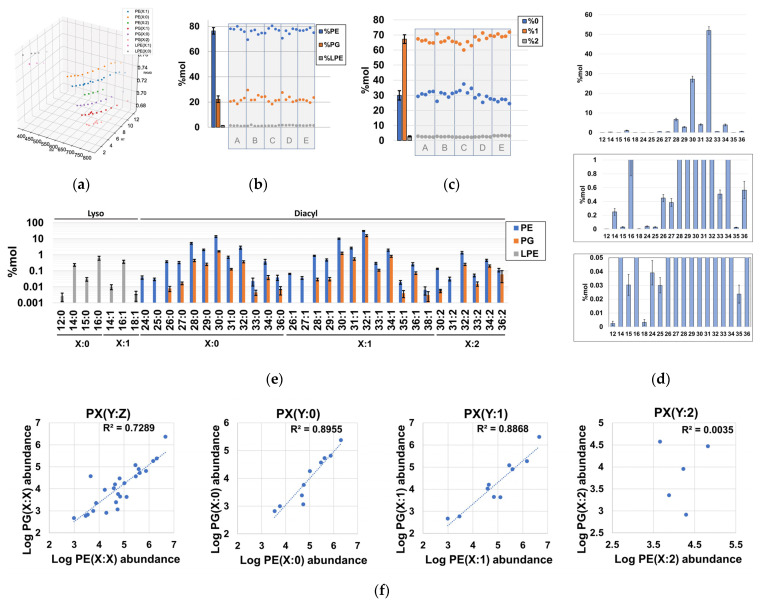
Qualitative and quantitative properties of sum compositions identified in the phospholipidome of *H. influenzae*. (**a**) three-dimensional Kendrick mass plot representing all chromatographic peaks corresponding to identified sum compositions; (**b**) %mol distribution of total PE, PG, and LPE with respect to the total phospholipid content; (**c**) %mol content of phospholipids according to their degree of unsaturation; (**d**) %mol distribution of sum composition carbon atoms in the total phospholipid content, irrespective of the unsaturation number; (**e**) %mol distribution of detected PEs, PGs, and LPEs according to their sum compositions. (**f**) Global- and series-dependent correlation of the logarithms of the abundances between PEs and PGs. Note that each dot represents the summation of the %mol attributable to species with identical sum composition, and population replicates were designated as A to E.

Using our lipidomics workflow and downstream extensive data curation, we determined that the phospholipidome of *H. influenzae* is composed of at least eight different lipid series, according to their polar head and unsaturation number (Figure 3a, Table S1). These lipid series corresponded to multiple GPs encompassed in 30-sum compositions of phosphatidylethanolamines (PEs), 25-sum compositions of phosphatidylglycerols (PG), and 7-sum compositions of lyso-phosphatidylethanolamines (LPE) (Tables S1 and S2). We observed that PEs accounted for the majority (~75%) of the phospholipid content, while ~20% of the total phospholipid content was attributable to PGs (Figure 3b, Table S7). These results were consistent with previous TLC studies [32] and proved the validity of our analysis. Interestingly, we identified LPE as a novel lipid subclass not previously described in *H. influenzae*, and it contributed to a very small fraction of the phospholipid content (Figure 3a,b, Table S7). Consistent with the literature, no other major lipid classes were identified in the phospholipid content of *H. influenzae*, demonstrating that this organism possesses one of the simplest phospholipidomes among gram-negative bacteria. However, lyso-phosphatidylglycerols and other phospholipids, such as phosphatidylserines, phos-phatidylglycerophosphates, phosphatidic acids, and their lyso-forms, may exist at pools with abundances below our instrumental limit of detection (Table S3) [18,32]. Regarding constituents of the lipidome other than phospholipids, we confidently annotated MG(18:0/0:0) and MG(0:0/18:0), as well as heptanoic acid (Table S1).

Evaluation of the unsaturation number distribution revealed that diunsaturated diacyl-GPs were minor components (Figure 3c), whereas the quantitative distribution of phospholipid-related sum compositions revealed that the carbon distribution in *H. influenzae* is qualitatively complex, quantitatively spanning at least ~4 orders of magnitude (Figure 3d,e, Table S7). Despite the relatively high number of sum compositions found for some lipid series (Figure 3e), it was determined that diacyl-GPs bearing 30 and 32 carbon units accounted for ~80% of the total phospholipidome content (Figure 3d). In particular, PE(28:0), PE(30:0), PE(30:1), and PE(32:1) exhibited very high abundances (Figure 3e, Table S7), which aligns with the total fatty acid composition determined via TLC, followed by GC-MS analysis of hydrolyzed phospholipid extracts [36]. However, the very high levels of PG(32:1) were not consistent with the fatty acyl compositions estimated for PGs, as no unsaturated acyl chains were reported [36] (Figure 3e). Notably, we observed a strong positive correlation between PE and PG logarithms of abundances in both saturated and monounsaturated phospholipids (Figure 3f, Table S1), which indicated that the processes governing the acyl chain distribution of PE and PG species in terms of order of magnitude are largely independent of the polar head in *H. influenzae*.

### 2.4. Fragmentation Analysis of Phospholipid Coelutions Allow for a Detailed Estimation of the Fatty Acyl Composition in the H. influenzae Lipidome and Positional Information of Fatty Acyl Chains in Diacylglycerophospholipids

**Figure 4:**
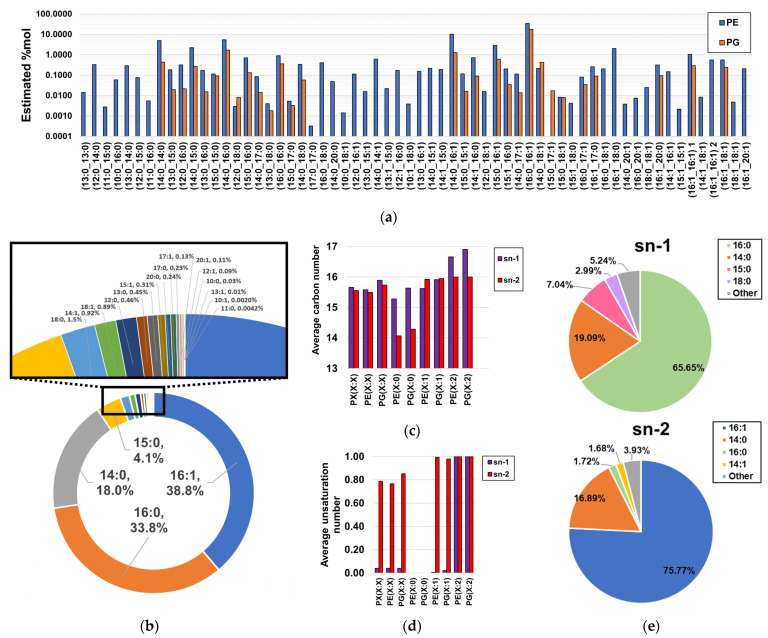
Estimated fatty acyl composition of the subset of fragmented sum compositions. (**a**) Estimated relative composition of lipid chain isomers; (**b**) proportion of fatty acyl chains bound to GPs; (**c**) length estimations of fatty acyl chains bound to the sn-1 and sn-2 positions of PEs and PGs; (**d**) unsaturation number estimations of fatty acyl chains bound to the sn-1 and sn-2 positions of PEs and PGs; (**e**) global estimations of fatty acyl composition found at both sn-1 and sn-2 positions of diacyl-GPs.

MS/MS analysis of molecular species coeluting with the same sum compositions allowed us to obtain the compositional information in fatty acyl chain content for ~96.9%mol of the detected phospholipidome (100%, ~99.5%, and ~95.2% of the %mol attributable to LPEs, PGs, and PEs, respectively, as shown in Tables S1 and S2), as fatty acyl information of coelutions corresponding to low-abundant sum-compositions could not be obtained (Table S1). Within this subset of the total phospholipid content, spectral evidence of 61 PEs and 29 PGs was found (Figure 4a, Table S2). Notably, it was found that the most abundant PEs and PGs contained C14:0, C16:0, C16:1, and C18:0 fatty acyl chains, as evidenced based on the estimated proportion of PE- and PG-bound fatty acids. In terms of consistency, only C14:0, C16:0, C16:1, and C18:0 were previously reported as phospholipid-bound fatty acyl residues in *H. influenzae* [36]. Here, our findings revealed the existence of a wide panel of low-abundant fatty acyl chains ranging from C10 to C20 and bearing a maximum of one unsaturation (Figure 4b), with the exception of C11:1, C19:0, and C19:1, suggesting that limitations in methodological sensitivity did not allow the detection of very low-level species in previous studies [36]. Interestingly, MS/MS fragmentation analysis of distant PE(32:2) peaks suggested a mixed profile of two diunsaturated PE types, as identical fatty acyl chains were observed at different RTs (Figure S1). We propose that this effect can be attributed to the coexistence of a mixed population of PE(X:2) bearing fatty acyl chains with unsaturated Z bonds and cyclopropane rings, despite conventional MS/MS fragmentation analysis being unable to distinguish between these isomers (Figure S1). Although diacyl-GP regioisomers were not separated via the analysis, we estimated the abundance-corrected carbon and unsaturation number of phospholipids by assuming that only the predominant regioisomer was present, an assumption that was in agreement with bacterial biological constraints for saturated and monounsaturated diacyl-GPs [40,41]. Under this assumption, it was observed that the sn-2 positions of saturated PEs and PGs bear notably shorter fatty acyl chains than those bound to the sn-1 position, while diunsaturated PEs and PGs exhibited the opposite effect (Figure 4c). Unsaturated fatty acyl chains were found to be preferentially bound to the sn-2 position of monounsaturated PEs and PGs (Figure 4d), which is consistent with the literature [40,41]. Notably, it was observed that these differences in fatty acyl chain binding to the sn-1 and sn-2 positions were principally attributable to the notable enrichment of C16:0 at the sn-1 position, as well as C16:1 at the sn-2 position (Figure 4e).

### 2.5. Probabilistic Rules Are a Major Factor Defining the Order of Magnitude of Lipid Species, Allowing the Prediction of Low-Level Species, Expansion and Refinement of the Phospholipidome

**Figure 5:**
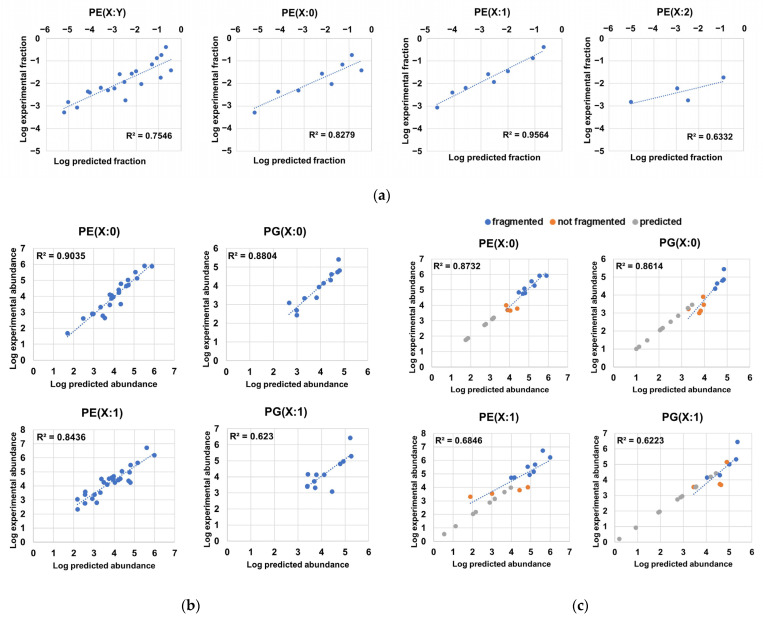
Predictability of compositional properties in *H. influenzae*. (**a**) Correlations between the logarithms of scaled abundance fractions of PEs and the logarithms of predicted scaled abundance fractions inferred from the sum of the pairwise product of LPE abundances containing fatty acyl residues compatible with the respective PE sum composition; (**b**) correlations between the logarithms of the experimental abundances and the logarithms of the predicted abundances of phospholipid chain isomers corresponding to PE(X:0), PE(X:1), PG(X:0). and PG(X:1) series; (**c**) correlations between the logarithms of the experimental abundances and the logarithms of the predicted abundances of sum compositions and superimposition of predicted sum compositions for PE(X:0), PE(X:1), PG(X:0), and PG(X:1) series. We note that most predicted abundances for sum compositions not measured in the analysis were below the lowest abundance corresponding to a sum composition within a lipid series, while predicted logarithm of abundance values in sum compositions absent in experimental data were additionally used in the y-axis to facilitate visualization.

Through the examination of the associations between the different constituents of the phospholipidome and their distribution patterns, we observed that highly abundant LPEs were associated with highly abundant PE sum compositions, as these elements encompassed PE chain isomers bearing fatty acyl chains that were also found in highly abundant LPEs (Figure 3e). After formally testing of this relationship, we found high correlation coefficients between the logarithms of experimentally determined abundance fractions, which corresponded to sum compositions in PE(X:0) and PE(X:1) series, and the logarithms of the predicted abundance fractions obtained via summing the pairwise and compatible abundance products of LPEs (Figure 5a, Table S8). Although the predictability of PE(X:2) sum composition abundances determined via this approach was modest (Figure 5a), these results suggest that LPEs are generated via PE hydrolysis based on the putative action of phospholipases without any significant overall preference for fatty acyl chains. Furthermore, this result suggests that the probability distributions of PE(X:0) and PE(X:1) series could be inferred from LPE abundances in terms of order of magnitude (Figure 5a). However, structural elucidation limited the inference of the probability distribution of LPEs from experimental PE abundance data using the opposite approach, as multiple PE chain isomers bearing fatty acyl chains not detected in LPE series coeluted and had equivalent PE sum compositions (Tables S1 and S3).

Based on the relationships between monoacyl- and diacyl-glycerophospholipids and between the observed experimental lipid chain isomer distribution (Figure 4a) and the total phospholipid-bound fatty acyl content (Figure 4b), we hypothesized that the distribution profile of PEs and PGs arises from the conditional probabilities associated with the unique probabilities of observing a fatty acyl chain in a given phospholipid. Inference of these probabilities from experimental data (Table 3) allowed us to determine very high correlation coefficients between the experimental and predicted logarithms of the abundances corresponding to saturated PE and PG species, suggesting that positional independence largely dominates the PE(X:0) and PG(X:0) profiles (Figure 5b, Table S9). In addition, the utility of the predictions was further confirmed through adequate estimation of the logarithms of abundances corresponding to sum compositions that were not fragmented and, thus, not used for the calculation of probabilities (Figure 5c). Interestingly, most of the undetected X:0 sum compositions that were predicted fell below the limit of detection (Figure 5b, Table S9). Thus, the model successfully predicted the order of magnitude of species that were undetectable via our MS method. In particular, we predicted the existence of 31 additional PE(X:0)s and 43 additional PG(X:0)s (Table S9). Analysis of the predictability of lipid chain isomer abundances in monounsaturated PEs and PGs revealed weaker correlation coefficients (~0.84 and ~0.62, respectively) (Figure 5b). Several possible explanations for this effect exist, including the following: (i) the calculation of fatty acyl chain probabilities in PX(Y:1) series was made via substitution taking scaled PX(Y:0) fatty acyl chain probabilities, as these figures could not be directly inferred from experimental data in PX(Y:1) due to the intrinsic nature of unsaturated diacyl-GPs; (ii) the partly resolved chromatographically species found in PX(Y:1) series may limit the accurate estimation of abundances for species within a definite sum composition; (iii) lipid chain isomers in PX(Y:1) bearing Z bonds or cyclopropane rings could follow distinct distribution probabilities; and (iv) PGs had significantly lower abundances, while given the high correlation found between PE- and PG-associated abundances (Figure 3f), individual fatty acyl chain probabilities calculated for PEs were used to predict PG abundances when these probabilities were unsolvable within PG data. This issue significantly constrained the number of fatty acyl chain probabilities available for PG(X:1) and, therefore, may restrict the predictability of the PG(X:1) series. Nonetheless, due to the relatively high correlation coefficient found between experimental and predicted PE(X:1) chain isomer abundance data (Figure 5b), we could predict the existence of 61 additional PE(X:1) chain isomers (Table S9). As expected (Figure 3f, Figure 5a), we observed low predictive capabilities for the compositional profile of lipids belonging to the PE(X:2) series and insufficient data to evaluate the abundance predictability of PG(X:2) series (Table S9). The modest results observed when evaluating the PE(X:2) series (Table S9) may be attributed to the following potential causes: (i) the low number of detected PE(X:2) sum compositions, (ii) the low overall abundance for PE(X:2) sum compositions, and (iii) the putative presence of a mixed population of PE(X:2) with different compositional behaviors due to the presence of Z bonds and cyclopropane rings in fatty acyl chains (Figure 1A, Figure S1). In sum, we considered the predictability of the compositional profile of PE(X:0), PG(X:0) and PE(X:1) chain isomers to be good enough to expand the number of diacyl-GP chain isomers by more than two-fold (135 predicted lipid chain isomers, in addition to the 90 chain isomers that were experimentally determined, as shown in Tables S2 and S9). Nevertheless, we suggest that deep lipidomics methodologies and computational optimization of probabilities may be employed to evaluate the positional independence and compositional properties of PG(X:1), PE(X:2), and PG(X:2) to generate more refined probabilistic models.

## 3. Materials and Methods

### 3.1. Reagents and Solutions

All solvents and reagents used in this study were of MS-grade quality. The polar extraction solution was 17.17 µM 4-chloro-phenylalanine (internal standard, Sigma-Aldrich, Steinheim, Germany) in Milli-Q water. The lipophilic extraction solution was prepared by adding methyl tert-butyl ether (Sigma-Aldrich, Steinheim, Germany) to MeOH (Thermo Fisher Scientific, Loughborough, UK) up to a 1:1 (*v*/*v*) ratio.

The CE-TOF/MS sample buffer solution was prepared by dissolving 0.2 mM methionine sulfone (internal standard, Sigma-Aldrich, Steinheim, Germany) in Milli-Q water containing 0.1 M formic acid (Sigma-Aldrich, Steinheim, Germany). The CE-TOF/MS sheath liquid solution was prepared by mixing 100 mL of Milli-Q water with 100 mL of 100% MeOH, 4 µL of concentrated formic acid, 10 µL of 5 mM purine, and 10 µL of 2.5 mM hexakis (1*H*,1*H*,3*H*-tetrafluoropropoxy)phosphazene HP722 (CE-TOF/MS reference masses, Agilent Technologies, Santa Clara, CA, USA). The CE-TOF/MS background electrolyte solution consisted of 1 M formic acid in MeOH:H_2_O 1:9 (*v*/*v*).

For LC-QTOF/MS, the mobile phase eluent A was 10 mM ammonium acetate (Fluka, Busch, Switzerland) and 0.2 mM ammonium fluoride (Sigma-Aldrich, Steinheim, Germany) in 9:1 H_2_O:MeOH (*v*/*v*). The mobile phase eluent B was 10 mM ammonium acetate and 0.2 mM ammonium fluoride in a mixture of 2:3:5 ACN:MeOH:isopropanol (*v*/*v*, ACN and isopropanol from Thermo Fisher Scientific, Loughborough, UK). The reference mass solution was 5% (*v*/*v*) Milli-Q water in ACN, as well as containing three reference masses (hypoxanthine, ammonium trifluoroacetate and (1H,1H,3H-tetrafluoropropoxy)phosphazene HP-0921) to allow correction and high mass resolution in MS.

For GC-QTOF/MS, the methoxymation solution was pyridine containing 15 mg·mL^−1^ O-methoxyamine (Sigma-Aldrich, Steinheim, Germany). The silylation solution was N,O-bis(trimethylsilyl)trifluoroacetamide (BSTFA) with 1% trimethylchlorosilane (TMCS) (Sigma-Aldrich, Steinheim, Germany). The GC/MS fatty acid methyl ester mix solution was prepared by diluting the grain fatty acid methyl esters mix (C8:0-22:1, Sigma-Aldrich, Steinheim, Germany) with dichloromethane (Carlo Erba Reagents, Sabadell, Spain) at a 1:100 (*v*/*v*) ratio. The GC/MS n-alkane mix was prepared by diluting the grain C8:C40 Alkane Calibration Standard (Sigma-Aldrich, Steinheim, Germany) with dichloromethane at a 1:5 ratio (*v*/*v*). The GC/MS internal standard solution was 71.8 µM tricosane (internal standard, Sigma-Aldrich, Steinheim, Germany) in n-heptane (GC grade, Panreac, Castellar del Vallès, Spain).

### 3.2. Bacterial Strains and Culture Conditions

*H. influenzae* RdKW20 (NC_000907.1) was cultured via continuous shaking (125 rpm) at 37 °C under microaerophilic conditions (5% CO_2_) in Haemophilus Test Medium (HTM) broth (Francisco Soria Melguizo S.A., Madrid, Spain). Five biological replicate populations of *H. influenzae* Rd KW20 were evolved for 10 days (approximately 100 generations, designated as A to E). Every 24 h, the grown cultures were diluted at a 1:1000 ratio (*v*/*v*) in fresh media (2 mL HTM). Five biological replicates were generated from each evolution replicate (total *n* = 25). The bacteria were then incubated overnight under the above-mentioned conditions. Subsequently, the cultures were diluted at a ratio of 1:100 (*v*/*v*) with fresh HTM and cultured until OD_600_ = 0.6. The bacterial pellets for mass spectrometry analysis were obtained via centrifugation (108× *g*; T = R.T.; t = 3 min), followed by washing with 800 µL PBS and another round of centrifugation (108× *g*; T = R.T.; t = 3 min). Next, 200 µL of cold methanol (−20 °C) was added to the pellets, followed by storage at (−80 °C) to minimize metabolism before quenching.

### 3.3. Cellular Lysis and Metabolite Extraction

To enhance metabolite recovery, double extraction was performed. The first polar extract was generated by adding 140 µL of polar extraction solution to the bacterial pellets preserved in cold MeOH (achieving a H_2_O:MeOH 1:1.43 (*v*/*v*) solvent mixture). Bacterial lysis was achieved through alternating ultrasonication cycles (20 pulses for 0.5 s each, amplitude = 60%) using a UP 200S ultrasonicator equipped with an S2 probe (Dr Hielscher GmbH, Stahnsdorf, Germany) with immersion in liquid N_2_ (t = 50 s). This process was repeated 20 times for each sample. After metabolite extraction on ice (t = 15 min), the samples were centrifuged (12,600× *g*; T = 4 °C, t = 30 min). The supernatant was aliquoted for further CE-TOF/MS and GC-QTOF/MS analyses. After collecting the supernatant, 200 µL of lipophilic extraction solution was added to the remaining pellet, which was then resuspended through ultrasonication (3 pulses for 0.5 s each, amplitude = 20%). The samples were kept on ice to prevent overheating (t = 1 min), followed by vortexing (T = R.T., t = 30 min), and placed at RT (t = 15 min) for metabolite extraction. The samples were centrifuged (16,300× *g*, T = R.T., t = 30 min), and 100 µL of the supernatant was transferred to LC/MS vials for LC-QTOF/MS analysis.

### 3.4. LC-QTOF/MS Analysis and Data Processing

MeOH:MTBE extracts were analyzed following an adapted version of the Agilent MassHunter Workstation Lipid Annotator method for lipidomics analysis described by Agilent Technologies [62]. The analysis was performed using an HPLC system (1200 series, Agilent Technologies, Santa Clara, CA, USA) coupled to an Agilent 6545 QTOF/MS analyzer (Agilent Technologies, Santa Clara, CA, USA). The injection volumes were 2 μL and 5 μL for positive and negative electrospray ionization (ESI) modes, respectively. Metabolites were separated via an Agilent InfinityLab Poroshell 120 EC-C18 column (3.0 mm × 100 mm, 2.7 μm; Agilent Technologies, Santa Clara, CA, USA) equipped with an Agilent InfinityLab Poroshell 120 EC-C18 guard column (3.0 mm × 5 mm, 2.7 μm; Agilent Technologies, Santa Clara, CA, USA). The column temperature was 50 °C. A mobile phase flow rate of 0.6 mL·min^−1^ was maintained throughout the chromatographic gradient. Firstly, 70% B was held until 1 min. Secondly, 86% B was achieved at 3.5 min and held until 10 min. Next, 100% B was achieved at 11 min and held until 17 min, followed by 2 min of re-equilibration time at 70% B. The total method runtime was 19 min. Metabolites were ionized using an ESI source with a nebulizer at 50 psi, a drying gas temperature of 200 °C a drying gas flow rate of 10 L·min^−1^, a sheath gas temperature of 300 °C, and a sheath gas flow rate of 12 L·min^−1^. In both polarity modes (ESI+ and ESI−), the capillary, fragmentor, skimmer, and 18ctupole radiofrequency voltages were set to to 3500, 150, 65, and 750 V, respectively. Firstly, full MS was selected as the data acquisition mode at an acquisition rate of 3.5 spectra·s^−1^ over a mass range of *m*/*z* 50 to 3000. Mass correction was performed using *m*/*z* 121.0509 and 922.0098 for ESI+ and *m*/*z* 112.9856, 980.0164, and 1033.9881 for ESI-mode. Next, two iterative auto MS/MS analyses (under both polarity modes) were conducted under identical chromatographic conditions. MS/MS spectra were systematically acquired via iterative MS/MS mode over subsequent injections from a sample pool. Isolation width, ramped collision energy, and offset values were set at ≈1.3 Da, 3.8, and 4.6, respectively. Additional iterative MS/MS analyses were performed by increasing the injection volume in both ESI polarities to increase the spectral quality of low-abundant features (injection volumes of 7 μL and 10 μL for ESI+ and ESI-, respectively). Data deconvolution, chromatogram alignment, and compound integration were performed using Agilent MassHunter Workstation Software Profinder (B.10.00, Agilent Technologies, Santa Clara, CA, USA). Kendrick masses were calculated by adjusting for CH_2_, enabling the generation of Kendrick mass diagrams for the systematic search of lipid series. We considered as a lipid series at least three molecular features of similar Kendrick mass defect, which were spaced by 14 mass units. Initial lipid series annotation was performed by merging the manually curated Kendrick Mass output with the automated annotations obtained from processing MS/MS files with Agilent MassHunter Workstation Lipid Annotator (v. 1.0, Agilent Technologies, Santa Clara, CA, USA) (precursor and fragment ppm tolerance = 20). Unannotated molecular features putatively belonging to annotated lipid series were annotated using CEU Mass Mediator Batch search [63]. A subsequent metabolite annotation curation pipeline was performed for annotations obtained via non-targeted metabolomics data reprocessing using the following steps: (i) annotations were confirmed based on the presence of selective diagnostic ions and neutral mass losses in the MS/MS spectra of lipids acquired via both ESI+ and ESI− modes; (ii) annotations were further curated by requiring the presence of a consistent adduct profile within lipid series ([M+H]^+^ > [M+Na]^+^ > [M+K]^+^, [M+H]^+^ > [M+NH_4_]^+^, and [M−H]^−^ > [M+CH_3_COO]^−^ for both PE and LPE series; [M+NH_4_]^+^ > [M+H]^+^, [M+Na]^+^ > [M+H]^+^, [M−H]^−^ > [M+CH_3_COO]^−^ for PG series); (iii) annotations of PX(Y:Z) (PX(Y:Z) was a generic GP, and X was defined as a polar head, Y was defined as a sum composition carbon number, and Z was defined as a sum composition unsaturation number) were consistent with a logical elution order (RT_PX(Y:0)_ > RT_PX(Y:1)_ > RT_PX(Y:2)_ at equal C number; RT_PG(Y:Z)_ < RT_PE(Y:Z)_ at equal C and unsaturation numbers; RT_PX(Y1:Z)_ > RT_PX(Y2:Z)_ if Y_1_ > Y_2_, at equal unsaturation number); (iv) manual inspection of extracted ion chromatograms for all predicted *m*/*z* corresponding to all PG, PE, and LPE lipid series, which contained a range of fatty acyl chains of C10:0–C20:0 and C10:1–C20:1, as no *m*/*z* corresponding to fatty acyl chains with C number lower than 10 or higher than 20 were observed in initial MS/MS data curation, and fatty acyl chains contained in GPs were determined to bear a maximum of one unsaturation. The curated set of annotations was used to re-integrate the LC-ESI(-)-QTOF/MS data from the [M−H]^−^ adducts of peaks that corresponded to the identified series LPE(X:0), LPE(X:1), PE(X:0), PE(X:1), PE(X:2), PG(X:0), PG(X:1), and PG(X:2) (X being a defined C number) using Agilent MassHunter Workstation Qualitative Analysis (B. 08.00, Agilent Technologies, Santa Clara, CA, USA) (*m*/*z* tolerance = 20 ppm). All annotations with more than one piece of orthogonal information (*m*/*z*, RT, MS/MS, adduct profile) were assigned as having an L2 confidence in the annotation. The chromatographic method was unable to resolve lipid chain isomers of saturated and isobaric diacyl-GPs and could only partially resolve lipid chain isomers of unsaturated and isobaric diacyl-GPs. Consequently, the area under the curve (AUC) of diacyl-GP lipid chain isomers and regioisomers (diacyl-GPs isomers bearing identical lipid chains but opposite binding to the sn-1 and sn-2 positions of their glycerol backbone) with identical sum composition that belonged to a specific phospholipid type (PE, PG, LPE) was summed for initial phospholipidome compositional analysis. Next, lipid chain isomers in each phospholipid coelution were determined based on the presence of compatible sums of *m*/*z* corresponding to fatty acyl chains in the collected MS/MS spectra. The predominant regioisomer in each lipid chain isomer was determined, as the relative intensity of the *m*/*z* generated from the fatty acyl chain bound to the sn-2 position of Pes and PGs was described as being higher with respect to that generated from the fatty acyl chain bound at sn-1 position [64]. LPE regioisomers with identical sum composition were also summed, as it was shown that artifactual interconversion occurs between these species [62,65]. Determination of the relative percentages of fatty acyl chains bound to GPs was performed using MS^1^-reprocessed data corresponding to LC-ESI(-)-QTOF/MS analyses. Firstly, raw phospholipid abundances of PGs and LPEs were normalized based on their relative response factor (Table S10) with respect to PE using data retrieved from the analysis of a mixture of 2.5 µL of SPLASH LipidoMIX™ Internal Standard (Avanti Polar Lipids, Alabaster, AL, USA) and 97.5 µL MeOH:MTBE 1:1 (*v*/*v*), which was analyzed under identical LC-ESI(-)-QTOF/MS conditions, as described above, in which the sample and QC MS^1^ data were acquired. Additional recursive metabolite annotation of LC-MS data was performed by manually searching for the presence of unidentified metabolites contained in *H. influenzae* metabolic models *i*CS400 and *i*NL638 by generating the expected *m*/*z* adducts [M+H]^+^, [M+NH_4_]^+^, [M+K]^+^, [M−H]^−^, [M+CH3COO]^−^ of metabolites present in the GEMs and manually evaluating the EIC of these predicted *m*/*z* in samples using Agilent MassHunter Workstation Qualitative Analysis (B. 08.00, Agilent Technologies, Santa Clara, CA, USA) (*m*/*z* tolerance = 20 ppm). RT being consistent with the predicted LogP metabolites present in *i*CS400 and *i*NL638 was also considered [66].

### 3.5. GC-QTOF/MS Analysis and Data Processing

Samples were derivatized and analyzed following a protocol adapted from a previously described methodology [67,68,69]. Firstly, 100 µL of each MeOH:H_2_O polar extract (1:1.43, *v*/*v*) were evaporated until dryness under high vacuum in a HyperVAC VC2124 vacuum concentrator (Gyrozen, Daejeon, Republic of Korea). Next, 10 µL of methoximation solution were added to the dried extracts, allowing methoximation of aldehyde and keto groups at R.T. (t = 16 h). Afterwards, 10 µL of silylation solution was added for trimethylsilylation of acid hydrogen-containing metabolites (t = 60 min; T = 70 °C). Vials were then cooled down (t = 30 min; T = R.T.) and reconstituted in 50 µL of GC/MS internal standard solution.

Sample analysis was performed in an Agilent 7890B GC system coupled to an Agilent 7250 accurate mass Q/TOF analyzer (both from Agilent Technologies, Santa Clara, CA, USA). Next, 2 µL of sample was injected into a multimode inlet at 250 °C with a 3:1 split ratio. Separation of metabolites then occurred using a capillary column (Agilent DB-5MS, 30 m × 0.25 mm, 0.25 µm film thickness; 95% dimethyl–5% diphenylpolysiloxane and 10 m guard column; Agilent Technologies, Santa Clara, CA, USA). The carrier gas (He) flow rate was set at 0.85 mL·min^−1^. The oven was allowed to stand at 60 °C for 1 min, and the temperature was gradually increased at 10 °C·min^−1^ until 325 °C, at which point it was held for 10 min. Metabolites were ionized using an electron ionization (EI) source. Full MS was selected as data acquisition mode, which used an acquisition rate of 6.67 spectra·s^−1^ over a mass range from *m*/*z* 45 to 650. D files acquired in profile mode were converted to the SureMass format to use the SureMass deconvolution algorithm from Agilent MassHunter Unknowns Analysis (B.10.00). The GC/MS fatty acid methyl ester described above was used to generate a correlation between Fiehn retention indexes (RI) and RTs. Similarly, the GC/MS n-alkane mix (C8–C40) was used to correlate NIST retention indexes with RTs. A spectral library search was initially performed for deconvoluted features using a combination of Exact Mass FiehnLib, Nominal Mass FiehnLib, and exact-mass in-house libraries of polyols, carbohydrates, and other polar compounds, all of which generated from data of pure standards analyzed via the same GC chromatographic method and using 70 eV in an EI source [67]. Hence, an L1 level of confidence in the annotation was assigned when match factor score > 70% or match factor < 70%, though the annotation was supported by acceptable RT error (RT %Error < 2%) and specific exact mass ions (File S1). An additional metabolite annotation was performed using the NIST Library (score cutoff > 80%), in which annotations were assigned an L2 confidence level if experimental RIs coming from experimental RTs using the n-alkane RI regression (RT %Error < 5%) were consistent with RIs collected in the NIST library. Metabolite annotations were manually curated, and qualifier and quantifier ions were selected based on their relative abundance and selectivity. Compound integration was performed using Agilent MassHunter Workstation Quantitative analysis for TOF (B.10.00, Agilent Technologies, Santa Clara, CA, USA). Additional recursive metabolite annotation over GC-MS data was performed by manually searching for the presence of metabolites contained in the *H. influenzae* metabolic models *i*CS400 and *i*NL638 in both Exact Mass and Nominal Mass FiehnLib. Annotations obtained from *i*CS400 and *i*NL638 were given an L1 level of confidence in the annotation when supported by RT tolerance (RT %Error < 2%) and characteristic exact mass ions (File S1). Qualifier and quantifier ions associated with these annotations were reintegrated using Agilent MassHunter Workstation Quantitative Analysis (B. 10.00, Agilent Technologies, Santa Clara, CA, USA).

### 3.6. CE-TOF/MS Analysis and Data Processing

Samples were treated and analyzed following the adaptation of a previously -described method [70]. In brief, 120 μL of H_2_O:MeOH extract was subjected to high vacuum on a HyperVAC VC2124 vacuum concentrator (Gyrozen, Daejeon, Republic of Korea) until complete dryness. Next, 60 µL of CE-TOF/MS sample buffer solution was added to each dried sample, which was vortexed for 1 min. After subsequent centrifugation (12,600× *g*; T = 4 °C; t = 15 min), the solution was transferred to CE/MS glass vials, which underwent centrifugation (4000× *g*; T = 4 °C; t = 20 min). Clear solutions were analyzed using a CE-TOF/MS platform composed of an Agilent 7100 CE system coupled to an Agilent 6224 TOF/MS analyzer (both from Agilent Technologies, Santa Clara, CA, USA). Samples were hydrodynamically injected at 50 mbar for 100 s and stacked by injecting background electrolyte at 100 mbar for 20 s. Metabolite separation was performed via a fused-silica capillary (70 cm, 50 μm i.d.; Agilent Technologies, Santa Clara, CA, USA). Separation conditions were +30 kV of capillary voltage (~2.4 mA) under normal polarity at 20 °C. Metabolites were ionized under ESI+ mode, with a sheath liquid flow of 6 µL·min^−1^, a nebulizer pressure of 10 psi, a drying gas temperature of 200 °C, and a flow rate of 10.0 L·min^−1^. Voltages for capillary, fragmentor, skimmer, and octopole were set to 5500, 125, 65, and 750 V, respectively. Full MS was selected as data acquisition mode, which was set at an acquisition rate of 1.36 spectra·s^−1^ over a mass range of *m*/*z* 70 to 1000. Mass calibrators *m*/*z* 121.0509 and 922.0098 were used for online mass correction. Data deconvolution, electropherogram alignment, and molecular feature integration were performed using Agilent MassHunter Workstation Software Profinder (B.10.00, Agilent Technologies, Santa Clara, CA, USA). Molecular features obtained using non-targeted metabolomics data reprocessing were subjected to initial metabolite annotation using the CE-MS Experimental RMT search from CEU Mass Mediator [71] (*m*/*z* tolerance = 20 ppm; RMT tolerance = 5%, except 10% for regions near the EOF or at very low migration times [71]), which uses data from an in-house library of pure standards run under identical method conditions. These annotations were assigned an L1 confidence in the annotation. Annotations of compounds not annotated in GC-MS were further supported based on the presence of characteristic in-source fragments [71]. Subsequently, unannotated molecular features were subjected to additional annotation using the CEU Mass Mediator Batch Search [63]. An L3 confidence in the annotation level was assigned for these annotations if compatible migration times with protonation status shown at separation pH (~2) were determined. Additionally, a recursive metabolite annotation over CE-MS data was performed by manually searching for the presence of metabolites contained in the *H. influenzae* metabolic models *i*CS400 and *i*NL638, thus extracting the predicted *m*/*z* from [M+H]^+^ adducts of metabolites using Agilent MassHunter Qualitative Analysis (B.08.00, Agilent Technologies, Santa Clara, CA, USA) (*m*/*z* tolerance = 20 ppm). Compatible features were subjected to the aforementioned annotation workflow for CE-TOF/MS and reintegrated using Agilent MassHunter Quantitative Analysis for TOF (B.10.00).

### 3.7. Curation and Obtention of the Small Molecule Set of iCS400 and iNL638 Metabolic Models

*i*CS400 [19] and *i*NL638 [18] *H. influenzae* GEMs were downloaded in the SMBL format from the BioModels repository (Model Identifiers MODEL1507180053 and MODEL2204040002). We did not use *i*JE296 [15], as this model was unavailable [18]. Next, metabolite IDs, names, and compartments for each model were extracted using Python (v. 3.10.1) and the COBRApy package (v. 0.26.3) [72], and they were exported into tabular format (File S3). KEGG Compound IDs and exact mass values for each compound were manually retrieved from the KEGG Compound database [73]. Both *i*CS400 and *i*NL638 were subjected to variable filtering, as only small molecules with defined mass <1500 Da were considered as ‘truly metabolites’ (entries corresponding to proteins, tRNAs, oligomers of ≥3 residues, and entries with MW ≥ 1500 Da were filtered out) (Table S3). To avoid biases in metabolite coverage estimation, only one representative example of each lipid type contained in models was retained for further global analysis of models (generic KEGG Compound IDs were assigned to these representative compounds) (Table S3).

### 3.8. Curation and Network Analysis of the Small Molecule Set of iCS400 and iNL638 Metabolic Models

The KEGG Compound IDs for annotated compounds found in GC-MS, LC-MS, and CE-MS were retrieved and curated manually from the KEGG Compound database [73]. Next, the information was crosschecked against the KEGG Compound IDs of the filtered small molecule datasets obtained from *i*CS400 and *i*NL638. Additionally, mapping of experimental metabolomics data was performed to a subnetwork of *i*NL638, which encompassed all periplasmic and cytoplasmic metabolites, as well as their associated reactions. This subnetwork was generated by importing *i*NL638 as a metabolite–reaction network using the cy3smbl App (v. 0.3.0) [74] in Cytoscape (v. 3.8.0) [75] and subsequently deleting the nodes not corresponding to metabolites or reactions, those corresponding to extracellular metabolites, and those of metabolic subproducts or cofactors with very high connectivities (H_2_O, CO_2_, AMP, ADP, ATP, NAD(P)(H), and H^+^) (File S2). The subnetwork was clustered using the GLay community clustering algorithm from the clusterMaker 2 (v. 2.0) Cytoscape App [76]. Functional annotation of clusters was conducted using KEGG Enrichment over network clusters using KOBAS-i, selecting *H. influenzae* Rd KW20 as the KEGG organism [77] (Table S6). The metabolome coverage of clusters was evaluated by considering only the total number of chemical species present in the filtered small molecule dataset obtained from *i*NL638, as described above. Over-representation analysis of clusters using the experimental metabolomics data as a query was performed through a Fischer exact test in each cluster using python (v. 3.10.1) and the scipy, seaborn, and math packages (File S4).

### 3.9. Global Evaluation of Metabolite Experimental Data

The chemical ontology of metabolites detected in experimental metabolomics data was determined using the ClassyFire algorithm [78]. The abundances of these metabolites were subjected to correlation analysis using MetaboAnalyst 5.0 [79]. Spearman’s rank correlation was performed over experimentally determined metabolites using default values, and correlation clusters with correlation coefficients of >0.6 and <−0.6 were subjected to further manual inspection. Enrichment of subnetwork clusters in features (variables) associated with self-correlating metabolite clusters was performed using python (v. 3.10.1) and the scipy, seaborn, and math packages (File S4).

### 3.10. Calculation of Experimental Lipidomic Properties

Response factor-corrected phospholipid abundances were converted to %mol. The total %mol content of each GP type (PE, PG, LPE) was determined for each measured sample extract. To evaluate the distribution of the total unsaturation number, we calculated the %mol of PX(Y:0), PX(Y:1), and PX(Y:2), respectively, which were summed for each sample extract. Similarly, the carbon number distribution of sum compositions was determined from C10 to C40, regardless of the unsaturation number. To calculate the fatty acyl average content bound to phospholipids, we first estimated the content of each given lipid chain isomer within an isobaric phospholipid coelution as the ratio between the sum of the intensity of the *m*/*z* associated with its two acyl chains and the total sum of intensities of combinations of *m*/*z* associated with fatty acyl chains compatible with the sum composition in the MS/MS spectra, as reported by Agilent MassHunter Lipid Annotator (v. 1.0, Agilent Technologies, Santa Clara, CA, USA), recognizing that the spectra were generated by combining multiple MS/MS spectra acquired via subsequent QC injections, followed by iterative auto MS/MS data acquisition, as described above for LC-ESI(-)-QTOF/MS. The entries corresponding to diacyl-GPs (Table S2) were selectively duplicated, and each fatty acyl chain was assigned to each of the generated entries. We then estimated the %mol corresponding to each bound fatty acyl chain. Metrics for sn-1 and sn-2 positions of PE and PG series were estimated by assuming that only the predominant regioisomer was present. To quantitatively assess the differences between the numbers of carbon atom and unsaturations, two parameters were calculated for the sn-1 and sn-2 position of each determined diacyl-GP chain isomer. These were named as ‘abundance-corrected average number of carbon atoms (Equation (1)), and ‘abundance-corrected average unsaturation number’ (Equation (2)), as shown in the corresponding equations,

(1) $$\mathrm{Abundance}-\mathrm{corrected}\;\mathrm{average}\;\mathrm{carbon}\;\mathrm{number}\;=\frac{\sum_{i=m}^{n}a_{i}\cdot c_{i}}{\sum_{i=m}^{n}a_{i}},$$

(2) $$\mathrm{Abundance}-\mathrm{corrected}\;\mathrm{average}\;\mathrm{unsaturation}\;\mathrm{number}=\frac{\sum_{i=m}^{n}a_{i}\cdot u_{i}}{\sum_{i=m}^{n}a_{i}},$$

where a represents the sum of abundances of lipid chain isomers containing a defined carbon atom number (Equation (1)) or a defined unsaturation number (Equation (2)) (m) from 1 to n, c represents a defined atom carbon number, and u represents a defined unsaturation number.

### 3.11. Calculation of Predictive Lipidomic Properties

We hypothesized that the probability of observing a defined PE or PG with *X_i_* and *X_j_* chains (*p*(*X_i_*_*X_j_*)) in *H. influenzae* is equal to the conditional probability of a PE or PG having specific acyl chains (*p*(*X_i_*_*X_j_*)) = *p*(*X_i_*|*X_j_*) = (*p*(*X_i_*) · *p*(*X_j_*)), as previously modelized for other biological matrices [80]. For the prediction of probability distributions of PEs from LPE composition, we assumed that *p*(*PE*(*X_i_*_*X_j_*)) = (*p*(*LPE*(*0*:*0*/*X_i_*)) + *p*(*LPE*(*X_i_*/*0*:*0*))) · (*p*(*LPE*(*0*:*0*/*X_j_*)) + *p*(*LPE*(*X_j_*/*0*:*0*))). To generate the predicted sum composition distribution, we then calculated *p*(*PE*(*X_i_*_*X_j_*)) for all possible pairwise combinations of fatty acyl chains contained in detected LPEs and summed all *p*(*PE*(*X_i_*_*X_j_*)) corresponding to lipid species with identical sum compositions (Table S8). For the prediction of PE and PG sum compositions and lipid chain isomers below the limit of detection from experimentally determined PE and PG data, we first considered the calculated *p*(*X_i_*_*X_j_*) for each experimentally determined PE or PG lipid chain isomer. Subsequently, we estimated *p*(*X_i_*) (*X* = *C10*:*0* to *C20*:*0*) in saturated PE and PG lipid series by first taking *p*(*X_i_*) as the square root of *p*(*X_i_*_*X_i_*) (for chain isomers with identical fatty acyl chains). Next, *p*(*X_i_*) that were not determinable via this method were solved via substitution that considered the *p*(*X_i_*_*X_j_*) of the lipid chain isomer of the same lipid series with the possible highest abundance and an already solved *p*(*X_j_*) other than *p*(*X_i_*). Given the high correlation observed between PE and PG abundances that corresponded to identical sum compositions and the lower abundance observed for *PG*(*X_i_*_*X_j_*) compared to equivalent *PE*(*X_i_*_*X_j_*), we used *p*(*X_i_*) values that were solved for PE series as *p*(*X_i_*) for fatty acyl chains when not determinable in the equivalent PG series. The *p*(*X_i_*) values were scaled for both saturated PE and PG series, meaning that $\overset{n}{\underset{i=m}{\sum }}p\left(X_{i}\right)=1$. For the prediction of *p*(*X_i_*) in monounsaturated PE and PG series, we used *p*(*X_i_*) obtained from saturated species and scaled them such as that $\overset{n}{\underset{i=m}{\sum }}p\left(X_{i},\;saturated\right)=0.5$, as the probability of randomly observing a saturated fatty acyl chain in a monounsaturated diacyl-GP was 0.5. Next, the *p*(*X_i_*, *unsaturated*) of monounsaturated fatty acyl chains were estimated through substitution. Lastly, probabilities from diunsaturated species were estimated using *p*(*X_i_*) values of monounsaturated PE and PG series, as only few diunsaturated PEs and PGs were observed, which did not allow a direct calculation of *p*(*X_i_*) from *p*(*X_i_*_*X_i_*) for low-level fatty acyl chains. All of the probabilities associated with each fatty acyl chain in each PE and PG series were used to generate all possible phospholipid chain isomer predicted abundances (Table S9). Predicted abundances of sum compositions were generated by summing all predicted abundances of lipid chain isomers that corresponded to the same sum composition (Table S9).

## 4. Conclusions

This study is the result of a combination of experimental findings originating from multiplatform metabolomics based on high-resolution analytical techniques, along with the development and application of fruitful mathematical models based on data and scientific principles. Consequently, we extended the direct evidence of the presence metabolome constituents of *H. influenzae*. Despite the relatively limited coverage of the metabolites predicted via GEMs, our established workflow for metabolome characterization enabled not only the relative quantitation, but also the identification, of lipid molecular species and polar metabolites not reported to date, of which the potential role of cyclo-(Leu-Pro) in *H. influenzae* pathogenesis is worth further investigation. We propose that analyzing correlating pools of metabolites could be a valuable resource for third-generation metabolic models that use metabolite-level information. Current metabolic models may be additionally benefit from the inclusion of the phospholipid composition that was elucidated through extensive study of the *H. influenzae* lipidome. Given the relative simplicity of the *H. influenzae* phospholipidome and its well-characterized genome, we foresee potential utility of *H. influenzae* as a model organism for exploring the factors that contribute to emergent membrane properties attributable to changes in phospholipid composition. Importantly, through the elucidation of the processes governing membrane composition in this relatively simple lipidome, we generated the first experimental in silico characterization of the phospholipidome. Further studies are needed to address the rules governing the compositional properties of membranes with higher lipid class diversity. Moreover, our study provides a mathematical rationale for the selective observation of specific lipid species, even though other species remain below the current instrumental limits of detection. We present the application to *H. influenzae* lipidome and beyond, proposing that revealing these processes could lead to more realistic membrane models and help in guiding the deep lipidome characterization of other biological specimens.

## Figures and Tables

**Table 1:** Pathway enrichment of *i*NL638 cytoplasmic and periplasmic subnetwork clusters, subnetwork cluster enrichment of the experimentally determined metabolome, and subnetwork cluster enrichment of metabolite groups corresponding to the identified self-correlation islands A–M.

Cluster Name	KOBAS-i Enriched Terms (pBH < 0.05)	Metabolite Cluster Enrichment Ratio	Cluster Enrichment pBH (Global Metabolome)	Cluster Enrichment (Correlation Islands, pBH < 0.05)
Cluster 1	Pyruvate metabolism, two-component system, alanine, aspartate and glutamate metabolism, citrate cycle (TCA cycle), oxidative phosphorylation, butanoate metabolism, phosphotransferase system (PTS), amino sugar and nucleotide sugar metabolism, sulfur metabolism, biosynthesis of amino acids, methane metabolism, 2-oxocarboxylic acid metabolism, cysteine and methionine metabolism, glycolysis/gluconeogenesis, arginine biosynthesis, pantothenate and CoA biosynthesis, valine, leucine and isoleucine biosynthesis, fructose and mannose metabolism, glycerophospholipid metabolism, and nitrogen metabolism.	1.868	0.094	K, L
Cluster 2	Thiamine metabolism and ABC transporters	0	1.000	-
Cluster 3	Starch and sucrose metabolism, amino sugar and nucleotide sugar metabolism, galactose metabolism, and glycolysis/gluconeogenesis.	2.163	1.000	-
Cluster 4	Folate biosynthesis, glyoxylate and dicarboxylate metabolism and methane metabolism.	0.541	1.000	-
Cluster 5	Pentose and glucuronate interconversions, fructose and mannose metabolism, pentose phosphate pathway, ascorbate and aldarate metabolism, glycolysis/gluconeogenesis, lipopolysaccharide biosynthesis, methane metabolism, biosynthesis of amino acids, and terpenoid backbone biosynthesis.	0.186	1.000	-
Cluster 6	Xanthosine and XMP transport and interconversions *.	0	1.000	-
Cluster 7	Purine metabolism, biosynthesis of amino acids, pyrimidine metabolism, alanine, aspartate and glutamate metabolism, histidine metabolism, phenylalanine, tyrosine and tryptophan metabolism, vitamin B6 metabolism, 2-oxocarboxylic acid metabolism, one carbon pool by folate, glutathione metabolism, arginine biosynthesis, glycine, serine and threonine metabolism, vancomycin resistance, and nicotinate and nicotinamide metabolism	1.287	0.827	J
Cluster 8	Sulfur relay system, folate biosynthesis, and ABC transporters.	0	1.000	-
Cluster 9	Valine tRNA-loading *	5.407	0.199	A
Cluster 10	Uridine and UMP transport and interconversions *.	1.352	1.000	D
Cluster 11	Pyrimidine metabolism, purine metabolism, ABC transporters, biotin metabolism, arginine biosynthesis, and sulfur relay system.	1.298	0.912	A
Cluster 12	Peptidoglycan biosynthesis, lysine metabolism, amino sugar and nucleotide sugar metabolism, biosynthesis of amino acids	1.202	1.000	-
Cluster 13	Fatty acid metabolism, fatty acid biosynthesis, propanoate metabolism, pyruvate metabolism, citrate cycle (TCA cycle), lysine degradation, biotin metabolism, sulfur metabolism, cysteine and methionine metabolism, butanoate metabolism, lipopolysaccharide biosynthesis, glycerolipid metabolism, and glycerophospholipid metabolism.	0.541	1.000	-
Cluster 14	Ubiquinone and other terpenoid–quinone biosynthesis, and terpenoid backbone metabolism.	0	1.000	-
Cluster 15	beta-Lactam resistance, peptidoglycan biosynthesis, ABC transporters, quorum sensing, and vancomycin resistance.	2.403	0.230	-
Cluster 16	Tyrosine metabolism *	3.605	0.296	E
Cluster 17	Fatty acid biosynthesis, fatty acid metabolism, and quorum sensing.	0	1.000	-
Cluster 18	ABC transporters and glutathione transport *.	1.992	0.127	B, F
Cluster 19	Glycerophospholipid metabolism, pyrimidine metabolism, pantothenate and CoA biosynthesis, glycine, serine and threonine metabolism, lipo-polysaccharide biosynthesis, terpenoid backbone biosynthesis, phenylalanine, and tyrosine and tryptophan metabolism	1.639	0.173	-
Cluster 20	One carbon pool folate, glutathione metabolism, selenocompound metabolism, and ABC transporters.	0.94	1.000	-
Cluster 21	Pyrimidine metabolism, purine metabolism and nicotinate and nicotinamide metabolism.	0	1.000	-
Cluster 22	Pantothenate and CoA biosynthesis, valine, leucine and isoleucine biosynthesis, biosynthesis of amino acids, arginine and proline metabolism, ABC transporters, glutathione metabolism, biosynthesis of secondary metabolism, 2-oxocarboxylic acid metabolism, C5-branched dibasic acid metabolism, butanoate metabolism, and citrate cycle (TCA cycle).	2.028	0.016	M
Cluster 23	Glycine, serine and threonine metabolism, biosynthesis of amino acids, lysine biosynthesis, cysteine and methionine metabolism, vitamin B6 metabolism, and 2-oxocarboxylic acid metabolism.	1.802	0.708	M
Cluster 24	Phenylalanine, tyrosine and tryptophan biosynthesis, and biosynthesis of amino acids.	0	1.000	-
Cluster 25	Selenocompound metabolism and aminoacyl-tRNA biosynthesis.	0	1.000	-
Cluster 26	Riboflavin metabolism, folate biosynthesis, pentose phosphate pathway, lipopolysaccharide biosynthesis, glutathione metabolism, and glyoxylate and dicarboxylate metabolism.	0.47	1.000	F
Cluster 27	Nicotinate and nicotinamide metabolism, purine metabolism, and pyrimidine metabolism.	2.704	0.044	I
Cluster 28	ABC transporters and ribose transport *.	3.605	0.169	I
Cluster 29	Purine metabolism, nicotinate and nicotinamide metabolism, and pyrimidine metabolism.	0	1.000	-
Cluster 30	ABC transporters and arginine biosynthesis.	3.004	0.023	F, K
Cluster 31	Purine metabolism, nitrogen metabolism	0	1.000	-
Cluster 32	ABC transporters and cationic antimicrobial peptide (CAMP) resistance.	0	1.000	-
Cluster 33	Biotin metabolism, fatty acid biosynthesis, and fatty acid metabolism.	0	1.000	-
Cluster 34	Aminoacyl-tRNA biosynthesis and phenylalanine transport *.	5.407	0.053	E
Cluster 35	Glycine, serine and threonine metabolism, glycolysis/gluconeogenesis, methane metabolism, biosynthesis of amino acids, glyoxylate and dicarboxylate metabolism, and glycerolipid metabolism.	2.317	0.184	B
Cluster 36	Glycerophospholipid metabolism, fatty acid biosynthesis, fatty acid metabolism, glycerolipid metabolism, and biotin metabolism.	0	1.000	-
Cluster 37	Glycerophospholipid metabolism and glycerolipid metabolism.	0	1.000	-
Cluster 38	Glycerophospholipid metabolism and glycerolipid metabolism.	0	1.000	-
Cluster 39	Glycerophospholipid metabolism and glycerolipid metabolism.	0	1.000	-
Cluster 40	Glycerophospholipid metabolism and glycerolipid metabolism.	0	1.000	-
Cluster 41	Fatty acid biosynthesis, fatty acid metabolism, biotin metabolism, glycerophospholipid metabolism, and glycerolipid metabolism.	0	1.000	-
Cluster 42	C5 branched dibasic acid metabolism, valine, leucine and isoleucine metabolism, 2-oxocarboxylic acid metabolism, biosynthesis of amino acids, and biosynthesis of secondary amino acids.	0	1.000	-
Cluster 43	Thiamine metabolism	0	1.000	-
Cluster 44	Pyrimidine metabolism and purine metabolism.	1.352	0.675	-
Cluster 45	CMP metabolism *	1.352	0.660	-
Cluster 46	dUMP metabolism *	0	1.000	-
Cluster 47	Inosine metabolism *	0	1.000	-
Cluster 48	ABC transporters and choline transport and incorporation into LOS *.	2.704	0.174	E
Cluster 49	Leucine transport and tRNA loading *.	5.407	0.037	M
Cluster 50	Histidine transport and tRNA loading.	3.605	0.095	E
Cluster 51	Glycerol and glyceraldehyde metabolism *.	1.545	0.397	C
Cluster 52	Biotin metabolism	0	1.000	-
Cluster 53	ABC transporters and arginine transport and tRNA loading *.	5.407	0.034	E

*: terms not identified via KOBAS-i but addressed through manual inspection of cluster nodes

**Table 2:** Identified polar metabolites absent in current developed GEMs.

Analytical Platform of Detection	Confidence in the Annotation	Matching Experimental Evidence	Metabolite Name
GC-QTOF/MS	L1	HRMS fragmentation spectra and retention times.	Cyclo(Leu-Pro)
			*N*-Methylalanine
			Cadaverine
			γ-Aminobutyric acid
			Thymine
			Cytosine
			Mannose
			Sucrose
			Trehalose
			Erythritol
			Ribitol
			Xylitol
			Sorbitol
	L2	Nominal mass fragmentation spectra and NIST retention indices.	Pseudouridine
			Threonic acid
CE-TOF/MS	L1	RMT, pseudomolecular ion *m*/*z* and in-source fragmentation.	Ophtalmic acid
			Betaine
			5’-Methylthioadenosine

HRMS: high-resolution mass spectrometry; RMT: relative migration time

**Table 3:** Estimated fatty acyl chain probabilities for each lipid series.

Fatty Acyl Chain	PE(X:0)	PG(X:0)	PE(X:1)	PG(X:1)	PE(X:2)	PG(X:2)
p10:0	0.015484	0.015072	0.007742	0.007536	0	0
p11:0	0.001413	0.001375	0.000706	0.000688	0	0
p12:0	0.036445	0.019686	0.018223	0.009843	0	0
p13:0	0.029268	0.042083	0.014634	0.021042	0	0
p14:0	0.552656	0.361674	0.276328	0.180837	0	0
p15:0	0.083368	0.166102	0.041684	0.083051	0	0
p16:0	0.233997	0.328652	0.116998	0.164326	0	0
p17:0	0.004429	0.011895	0.002214	0.005948	0	0
p18:0	0.037556	0.048219	0.018778	0.024109	0	0
p19:0	0	0	0	0	0	0
p20:0	0.005385	0.005241	0.002692	0.002621	0	0
p10:1	0	0	0.002384	0.003745	0.004768	0.00749
p11:1	0	0	0	0	0	0
p12:1	0	0	0.016958	0.026642	0.033916	0.053284
p13:1	0	0	0.006165	0.009686	0.01233	0.019371
p14:1	0	0	0.025861	0.024321	0.051722	0.048642
p15:1	0	0	0.009048	0.009295	0.018096	0.018589
p16:1	0	0	0.432158	0.312268	0.864315	0.624535
p17:1	0	0	0.0048	0.009216	0.0096	0.018432
p18:1	0	0	0.002466	0.104641	0.004932	0.209282
p19:1	0	0	0	0	0	0
p20:1	0	0	0.00016	0.000187	0.00032	0.000374

## Data Availability

Mass spectrometry data were uploaded to the Metabolomics Workbench Server (Dataset Identifier: ST002763).

## Supplementary Materials

The following supporting information can be downloaded at: https://www.mdpi.com/article/10.3390/ijms241311150/s1.

## References

[1] Agrawal A., Murphy T.F. (2011). Haemophilus influenzae infections in the *H. influenzae* type b conjugate vaccine era. J. Clin. Microbiol..

[2] LaClaire L.L., Tondella M.L., Beall D.S., Noble C.A., Raghunathan P.L., Rosenstein N.E., Popovic T., Members A.B.C.S.T. (2003). Identification of Haemophilus influenzae serotypes by standard slide agglutination serotyping and PCR-based capsule typing. J. Clin. Microbiol..

[3] Khattak Z.E., Anjum F. (2021). Haemophilus Influenzae. StatPearls [Internet].

[4] Wahl B., O’Brien K.L., Greenbaum A., Majumder A., Liu L., Chu Y., Lukšić I., Nair H., McAllister D.A., Campbell H. (2018). Burden of Streptococcus pneumoniae and Haemophilus influenzae type b disease in children in the era of conjugate vaccines: Global, regional, and national estimates for 2000–15. Lancet Glob. Health.

[5] Langereis J.D., de Jonge M.I. (2015). Invasive Disease Caused by Nontypeable Haemophilus influenzae. Emerg. Infect. Dis..

[6] Van Eldere J., Slack M.P., Ladhani S., Cripps A.W. (2014). Non-typeable Haemophilus influenzae, an under-recognised pathogen. Lancet Infect. Dis..

[7] Behrouzi A., Vaziri F., Rahimi-Jamnani F., Afrough P., Rahbar M., Satarian F., Siadat S.D. (2017). Vaccine Candidates against Nontypeable Haemophilus influenzae: A Review. Iran. Biomed. J..

[8] Poje G., Redfield R.J. (2003). General methods for culturing Haemophilus influenzae. Methods Mol. Med..

[9] Fleischmann R.D., Adams M.D., White O., Clayton R.A., Kirkness E.F., Kerlavage A.R., Bult C.J., Tomb J.F., Dougherty B.A., Merrick J.M. (1995). Whole-genome random sequencing and assembly of Haemophilus influenzae Rd. Science.

[10] Baddal B., Muzzi A., Censini S., Calogero R.A., Torricelli G., Guidotti S., Taddei A.R., Covacci A., Pizza M., Rappuoli R. (2015). Dual RNA-seq of Nontypeable Haemophilus influenzae and Host Cell Transcriptomes Reveals Novel Insights into Host-Pathogen Cross Talk. mBio.

[11] Link A.J., Hays L.G., Carmack E.B., Yates J.R. (1997). Identifying the major proteome components of Haemophilus influenzae type-strain NCTC 8143. Electrophoresis.

[12] Qu J., Lesse A.J., Brauer A.L., Cao J., Gill S.R., Murphy T.F. (2010). Proteomic expression profiling of Haemophilus influenzae grown in pooled human sputum from adults with chronic obstructive pulmonary disease reveal antioxidant and stress responses. BMC Microbiol..

[13] Kolker E., Purvine S., Galperin M.Y., Stolyar S., Goodlett D.R., Nesvizhskii A.I., Keller A., Xie T., Eng J.K., Yi E. (2003). Initial proteome analysis of model microorganism Haemophilus influenzae strain Rd KW20. J. Bacteriol..

[14] Post D.M., Held J.M., Ketterer M.R., Phillips N.J., Sahu A., Apicella M.A., Gibson B.W. (2014). Comparative analyses of proteins from Haemophilus influenzae biofilm and planktonic populations using metabolic labeling and mass spectrometry. BMC Microbiol..

[15] Edwards J.S., Palsson B.O. (1999). Systems properties of the Haemophilus influenzae Rd metabolic genotype. J. Biol. Chem..

[16] Othman D.S., Schirra H., McEwan A.G., Kappler U. (2014). Metabolic versatility in Haemophilus influenzae: A metabolomic and genomic analysis. Front. Microbiol..

[17] Tatusov R.L., Mushegian A.R., Bork P., Brown N.P., Hayes W.S., Borodovsky M., Rudd K.E., Koonin E.V. (1996). Metabolism and evolution of Haemophilus influenzae deduced from a whole-genome comparison with Escherichia coli. Curr. Biol..

[18] López-López N., León D.S., de Castro S., Díez-Martínez R., Iglesias-Bexiga M., Camarasa M.J., Menéndez M., Nogales J., Garmendia J. (2022). Interrogation of Essentiality in the Reconstructed Haemophilus influenzae Metabolic Network Identifies Lipid Metabolism Antimicrobial Targets: Preclinical Evaluation of a FabH β-Ketoacyl-ACP Synthase Inhibitor. mSystems.

[19] Schilling C.H., Palsson B.O. (2000). Assessment of the metabolic capabilities of Haemophilus influenzae Rd through a genome-scale pathway analysis. J. Theor. Biol..

[20] López-López N., Euba B., Hill J., Dhouib R., Caballero L.A., Leiva J., Hosmer J., Cuesta S., Ramos-Vivas J., Díez-Martínez R. (2020). Glucose Catabolism Leading to Production of the Immunometabolite Acetate Has a Key Contribution to the Host Airway-Pathogen Interplay. ACS Infect. Dis..

[21] Muda N.M., Nasreen M., Dhouib R., Hosmer J., Hill J., Mahawar M., Schirra H.J., McEwan A.G., Kappler U. (2019). Metabolic analyses reveal common adaptations in two invasive Haemophilus influenzae strains. Pathog. Dis..

[22] Ares-Arroyo M., Fernández-García M., Wedel E., Montero N., Barbas C., Rey-Stolle M.F., Garcia A., González-Zorn B. (2022). Genomics, Transcriptomics, and Metabolomics Reveal That Minimal Modifications in the Host Are Crucial for the Compensatory Evolution of ColE1-Like Plasmids. mSphere.

[23] May P., Wienkoop S., Kempa S., Usadel B., Christian N., Rupprecht J., Weiss J., Recuenco-Munoz L., Ebenhöh O., Weckwerth W. (2008). Metabolomics-and proteomics-assisted genome annotation and analysis of the draft metabolic network of Chlamydomonas reinhardtii. Genetics.

[24] Mouchlis V.D., Armando A., Dennis E.A. (2019). Substrate-Specific Inhibition Constants for Phospholipase A. J. Med. Chem..

[25] Rose T.D., Köhler N., Falk L., Klischat L., Lazareva O.E., Pauling J.K. (2023). Lipid network and moiety analysis for revealing enzymatic dysregulation and mechanistic alterations from lipidomics data. Brief. Bioinform..

[26] Tang X., Chang S., Qiao W., Luo Q., Chen Y., Jia Z., Coleman J., Zhang K., Wang T., Zhang Z. (2021). Structural insights into outer membrane asymmetry maintenance in Gram-negative bacteria by MlaFEDB. Nat. Struct. Mol. Biol..

[27] Bogdanov M., Pyrshev K., Yesylevskyy S., Ryabichko S., Boiko V., Ivanchenko P., Kiyamova R., Guan Z., Ramseyer C., Dowhan W. (2020). Phospholipid distribution in the cytoplasmic membrane of Gram-negative bacteria is highly asymmetric, dynamic, and cell shape-dependent. Sci. Adv..

[28] Nikaido H. (2003). Molecular basis of bacterial outer membrane permeability revisited. Microbiol. Mol. Biol. Rev..

[29] Schweda E.K., Richards J.C., Hood D.W., Moxon E.R. (2007). Expression and structural diversity of the lipopolysaccharide of Haemophilus influenzae: Implication in virulence. Int. J. Med. Microbiol..

[30] Mikhail I., Yildirim H.H., Lindahl E.C., Schweda E.K. (2005). Structural characterization of lipid A from nontypeable and type f Haemophilus influenzae: Variability of fatty acid substitution. Anal. Biochem..

[31] Sohlenkamp C., Geiger O. (2016). Bacterial membrane lipids: Diversity in structures and pathways. FEMS Microbiol. Rev..

[32] Sutrina S.L., Scocca J.J. (1976). Phospholipids of Haemophilus influenzae Rd during exponential growth and following the development of competence for genetic transformation. J. Gen. Microbiol..

[33] Murzyn K., Róg T., Pasenkiewicz-Gierula M. (2005). Phosphatidylethanolamine-phosphatidylglycerol bilayer as a model of the inner bacterial membrane. Biophys. J..

[34] Leekumjorn S., Cho H.J., Wu Y., Wright N.T., Sum A.K., Chan C. (2009). The role of fatty acid unsaturation in minimizing biophysical changes on the structure and local effects of bilayer membranes. Biochim. Biophys. Acta.

[35] Alberts B., Johnson A., Lewis J., Raff M., Roberts K., Walter P. (2014). Molecular Biology of the Cell.

[36] Fernández-Calvet A., Rodríguez-Arce I., Almagro G., Moleres J., Euba B., Caballero L., Martí S., Ramos-Vivas J., Bartholomew T.L., Morales X. (2018). Modulation of Haemophilus influenzae interaction with hydrophobic molecules by the VacJ/MlaA lipoprotein impacts strongly on its interplay with the airways. Sci. Rep..

[37] Jaisinghani N., Seeliger J.C. (2021). Recent advances in the mass spectrometric profiling of bacterial lipids. Curr. Opin. Chem. Biol..

[38] Cao W., Cheng S., Yang J., Feng J., Zhang W., Li Z., Chen Q., Xia Y., Ouyang Z., Ma X. (2020). Large-scale lipid analysis with C=C location and sn-position isomer resolving power. Nat. Commun..

[39] Jeucken A., Molenaar M.R., van de Lest C.H.A., Jansen J.W.A., Helms J.B., Brouwers J.F. (2019). A Comprehensive Functional Characterization of Escherichia coli Lipid Genes. Cell Rep..

[40] Zhang Y.M., Rock C.O. (2008). Membrane lipid homeostasis in bacteria. Nat. Rev. Microbiol..

[41] Seltmann G., Holst O. (2002). The Bacterial Cell Wall.

[42] Rakusanova S., Fiehn O., Cajka T. (2023). Toward building mass spectrometry-based metabolomics and lipidomics atlases for biological and clinical research. TrAC Trends Anal. Chem..

[43] Frainay C., Schymanski E.L., Neumann S., Merlet B., Salek R.M., Jourdan F., Yanes O. (2018). Mind the Gap: Mapping Mass Spectral Databases in Genome-Scale Metabolic Networks Reveals Poorly Covered Areas. Metabolites.

[44] Begum Ahil S., Hira K., Shaik A.B., Pal P.P., Kulkarni O.P., Araya H., Fujimoto Y. (2019). l-Proline-based-cyclic dipeptides from Pseudomonas sp. (ABS-36) inhibit pro-inflammatory cytokines and alleviate crystal-induced renal injury in mice. Int. Immunopharmacol..

[45] Zhai Y., Shao Z., Cai M., Zheng L., Li G., Yu Z., Zhang J. (2019). Cyclo(l-Pro–l-Leu) of Pseudomonas putida MCCC1A00316 Isolated from Antarctic Soil: Identification and Characterization of Activity against Meloidogyne incognita. Molecules.

[46] Li M., Huiru Z., Biting D., Yun J., Wei J., Kunming D. (2013). Study on the anti-quorum sensing activity of a marine bacterium Staphylococcus saprophyticus 108. BTAIJ.

[47] Parasuraman P., Devadatha B., Sarma V.V., Ranganathan S., Ampasala D.R., Reddy D., Kumavath R., Kim I.W., Patel S.K.S., Kalia V.C. (2020). Inhibition of Microbial Quorum Sensing Mediated Virulence Factors by. J. Microbiol. Biotechnol..

[48] Gowrishankar S., Sivaranjani M., Kamaladevi A., Ravi A.V., Balamurugan K., Karutha Pandian S. (2016). Cyclic dipeptide cyclo(l-leucyl-l-prolyl) from marine Bacillus amyloliquefaciens mitigates biofilm formation and virulence in Listeria monocytogenes. Pathog. Dis..

[49] Marchesan J.T., Morelli T., Moss K., Barros S.P., Ward M., Jenkins W., Aspiras M.B., Offenbacher S. (2015). Association of Synergistetes and Cyclodipeptides with Periodontitis. J. Dent. Res..

[50] Cerneckis J., Cui Q., He C., Yi C., Shi Y. (2022). Decoding pseudouridine: An emerging target for therapeutic development. Trends Pharmacol. Sci..

[51] Preumont A., Snoussi K., Stroobant V., Collet J.F., Van Schaftingen E. (2008). Molecular identification of pseudouridine-metabolizing enzymes. J. Biol. Chem..

[52] Thapa K., Oja T., Metsä-Ketelä M. (2014). Molecular evolution of the bacterial pseudouridine-5’-phosphate glycosidase protein family. FEBS J..

[53] Vergauwen B., Elegheert J., Dansercoer A., Devreese B., Savvides S.N. (2010). Glutathione import in Haemophilus influenzae Rd is primed by the periplasmic heme-binding protein HbpA. Proc. Natl. Acad. Sci. USA.

[54] Lin M.C., Wagner C. (1975). Purification and characterization of N-methylalanine dehydrogenase. J. Biol. Chem..

[55] Hamana K., Nakata K. (2000). Distribution of diaminopropane, putrescine and cadaverine in Haemophilus and Actinobacillus. Microbios.

[56] Erwin A.L., Nelson K.L., Mhlanga-Mutangadura T., Bonthuis P.J., Geelhood J.L., Morlin G., Unrath W.C., Campos J., Crook D.W., Farley M.M. (2005). Characterization of genetic and phenotypic diversity of invasive nontypeable Haemophilus influenzae. Infect. Immun..

[57] Fan X., Pericone C.D., Lysenko E., Goldfine H., Weiser J.N. (2003). Multiple mechanisms for choline transport and utilization in Haemophilus influenzae. Mol. Microbiol..

[58] Carmody J.M., Herriott R.M. (1970). Thymine and thymidine uptake by Haemophilus influenzae and the labeling of deoxyribonucleic acid. J. Bacteriol..

[59] Macfadyen L.P., Dorocicz I.R., Reizer J., Saier M.H., Redfield R.J. (1996). Regulation of competence development and sugar utilization in Haemophilus influenzae Rd by a phosphoenolpyruvate:fructose phosphotransferase system. Mol. Microbiol..

[60] Holt J.G., Krieg N.R., Sneath P.H.A., Staley J.T., ST W. (1994). Facultatively Anaerobic GramNegative Rods Subgroup 3: Genus Haemophilus. Bergey’s Manual of Determinative Bacteriology.

[61] Simpson G.L., Ortwerth B.J. (2000). The non-oxidative degradation of ascorbic acid at physiological conditions. Biochim. Biophys. Acta.

[62] Koelmel J., Sartain M., Salcedo J., Murali A., Xiangdong L., Stow S. Improving Coverage of the Plasma Lipidome Using Iterative MS/MS Data Acquisition Combined with Lipid Annotator Software and 6546 LC/Q-TOF. https://www.agilent.com/cs/library/applications/application-6546-q-tof-lipidome-5994-0775en-agilent.pdf.

[63] Gil-de-la-Fuente A., Godzien J., Saugar S., Garcia-Carmona R., Badran H., Wishart D.S., Barbas C., Otero A. (2019). CEU Mass Mediator 3.0: A Metabolite Annotation Tool. J. Proteome Res..

[64] Han X. (2016). Lipidomics: Comprehensive Mass Spectrometry of Lipids.

[65] Plückthun A., Dennis E.A. (1982). Acyl and phosphoryl migration in lysophospholipids: Importance in phospholipid synthesis and phospholipase specificity. Biochemistry.

[66] Cheng T., Zhao Y., Li X., Lin F., Xu Y., Zhang X., Li Y., Wang R., Lai L. (2007). Computation of octanol-water partition coefficients by guiding an additive model with knowledge. J. Chem. Inf. Model..

[67] Fiehn O. (2006). Metabolite profiling in Arabidopsis. Methods Mol. Biol..

[68] Rey-Stolle F., Dudzik D., Gonzalez-Riano C., Fernández-García M., Alonso-Herranz V., Rojo D., Barbas C., García A. (2022). Low and high resolution gas chromatography-mass spectrometry for untargeted metabolomics: A tutorial. Anal. Chim. Acta.

[69] Mastrangelo A., Ferrarini A., Rey-Stolle F., García A., Barbas C. (2015). From sample treatment to biomarker discovery: A tutorial for untargeted metabolomics based on GC-(EI)-Q-MS. Anal. Chim. Acta.

[70] López-Gonzálvez Á., Godzien J., García A., Barbas C. (2019). Capillary Electrophoresis Mass Spectrometry as a Tool for Untargeted Metabolomics. Methods Mol. Biol..

[71] Mamani-Huanca M., de la Fuente A.G., Otero A., Gradillas A., Godzien J., Barbas C., López-Gonzálvez Á. (2021). Enhancing confidence of metabolite annotation in Capillary Electrophoresis-Mass Spectrometry untargeted metabolomics with relative migration time and in-source fragmentation. J. Chromatogr. A.

[72] Ebrahim A., Lerman J.A., Palsson B.O., Hyduke D.R. (2013). COBRApy: COnstraints-Based Reconstruction and Analysis for Python. BMC Syst. Biol..

[73] Kanehisa M., Furumichi M., Tanabe M., Sato Y., Morishima K. (2017). KEGG: New perspectives on genomes, pathways, diseases and drugs. Nucleic Acids Res..

[74] König M., Dräger A., Holzhütter H.G. (2012). CySBML: A Cytoscape plugin for SBML. Bioinformatics.

[75] Shannon P., Markiel A., Ozier O., Baliga N.S., Wang J.T., Ramage D., Amin N., Schwikowski B., Ideker T. (2003). Cytoscape: A software environment for integrated models of biomolecular interaction networks. Genome Res..

[76] Utriainen M., Morris J.H. (2023). clusterMaker2: A major update to clusterMaker, a multi-algorithm clustering app for Cytoscape. BMC Bioinform..

[77] Bu D., Luo H., Huo P., Wang Z., Zhang S., He Z., Wu Y., Zhao L., Liu J., Guo J. (2021). KOBAS-i: Intelligent prioritization and exploratory visualization of biological functions for gene enrichment analysis. Nucleic Acids Res..

[78] Djoumbou Feunang Y., Eisner R., Knox C., Chepelev L., Hastings J., Owen G., Fahy E., Steinbeck C., Subramanian S., Bolton E. (2016). ClassyFire: Automated chemical classification with a comprehensive, computable taxonomy. J. Cheminform..

[79] Pang Z., Chong J., Zhou G., de Lima Morais D.A., Chang L., Barrette M., Gauthier C., Jacques P., Li S., Xia J. (2021). MetaboAnalyst 5.0: Narrowing the gap between raw spectra and functional insights. Nucleic Acids Res..

[80] Zarringhalam K., Zhang L., Kiebish M.A., Yang K., Han X., Gross R.W., Chuang J. (2012). Statistical analysis of the processes controlling choline and ethanolamine glycerophospholipid molecular species composition. PLoS ONE.

