# Supplementary figures and images for: Multiplatform Metabolomics Characterization Reveals Novel Metabolites and Phospholipid Compositional Rules of Haemophilus influenzae Rd KW20
Source: Int J Mol Sci. 2023 Jul 6;24(13):11150. doi: 10.3390/ijms241311150 (PMC10342370; doi:10.3390/ijms241311150)

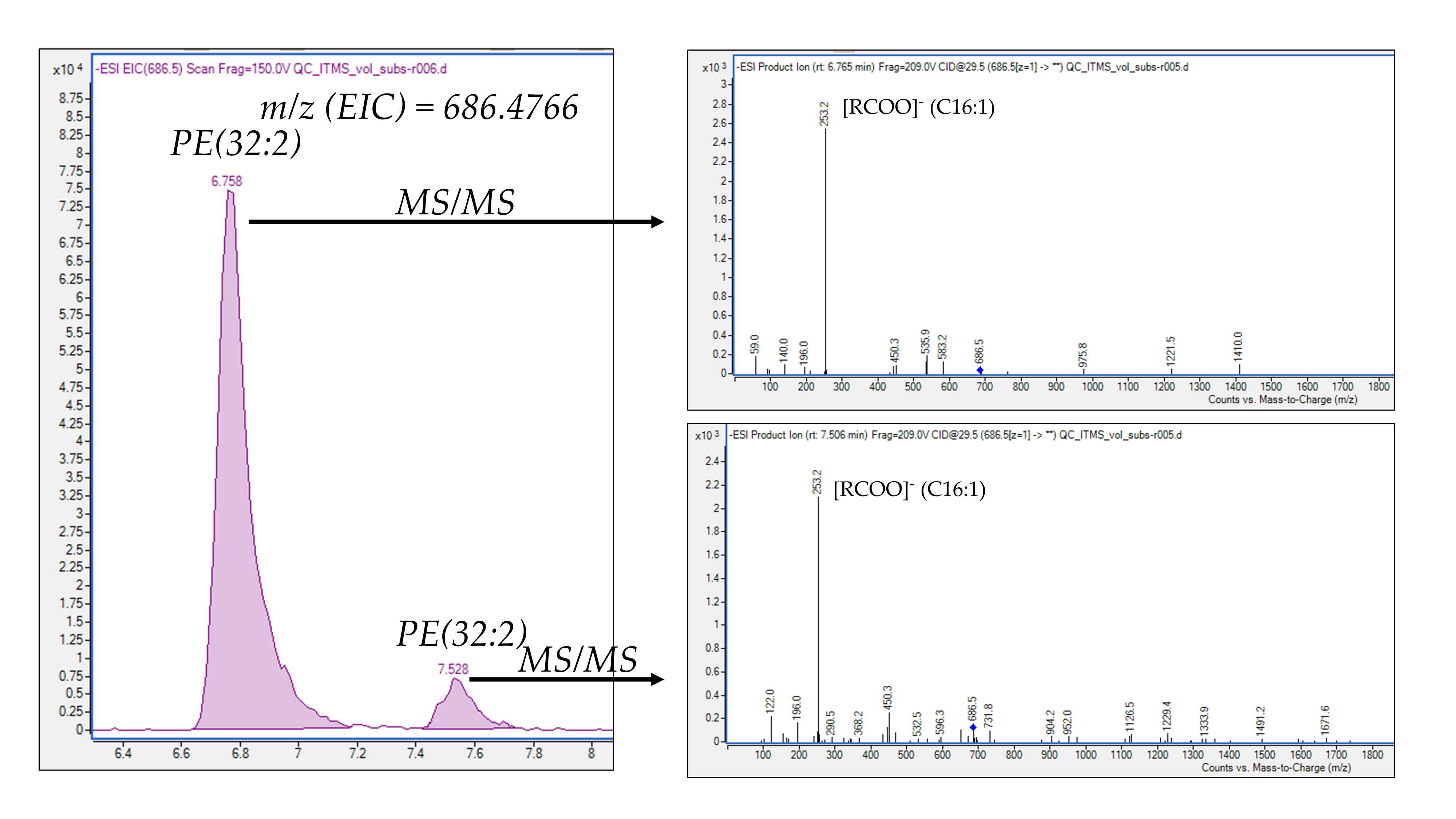

Supplement: Supplementary file 1 [file ijms-24-11150-s001.zip › Figure S1.tif]
